# Voluntary alcohol intake alters the motivation to seek intravenous oxycodone and neuronal activation during the reinstatement of oxycodone and sucrose seeking

**DOI:** 10.1038/s41598-023-46111-1

**Published:** 2023-11-06

**Authors:** Courtney S. Wilkinson, Harrison L. Blount, Shane Davis, Giselle Rojas, Lizhen Wu, Niall P. Murphy, Marek Schwendt, Lori A. Knackstedt

**Affiliations:** 1 Psychology Department, University of Florida, 114 Psychology, 945 Center Dr., Gainesville, FL 32611 USA; 2 Center for Addiction Research and Education, University of Florida, Gainesville, FL USA; 3 Orthodontics Department, University of Florida, Gainesville, FL USA

**Keywords:** Addiction, Motivation

## Abstract

Opioid-alcohol polysubstance use is prevalent and worsens treatment outcomes. Here we assessed whether co-consumption of oxycodone and alcohol influence the intake of one another, demand for oxycodone, and the neurocircuitry underlying cue-primed reinstatement of oxycodone-seeking. Male and female rats underwent oxycodone intravenous self-administration (IVSA) with homecage access to alcohol (20% v/v) and/or water immediately after the IVSA session. Next, economic demand for intravenous oxycodone was assessed while access to alcohol and/or water continued. Control rats self-administered sucrose followed by access to alcohol and/or water. Rats underwent a cue-primed reinstatement test and brains were processed for c-fos mRNA expression. While both sexes decreased oxycodone intake if they had access to alcohol, and decreased alcohol intake if they had access to oxycodone, only female oxycodone + alcohol rats exhibited decreased demand elasticity and increased cue-primed reinstatement. Alcohol consumption increased the number of basolateral and central amygdala neurons activated during sucrose and oxycodone reinstatement and the number of ventral and dorsal striatum neurons engaged by sucrose reinstatement. Nucleus accumbens shell dopamine 1 receptor expressing neurons displayed activation patterns consistent with oxycodone reinstatement. Thus, alcohol alters the motivation to seek oxycodone in a sex-dependent manner and the neural circuitry engaged by cue-primed reinstatement of sucrose and oxycodone-seeking.

## Introduction

From 2002 to 2012 there was a 15-fold increase in the prevalence of persons with alcohol use disorder (AUD) also reporting opioid use disorder (OUD), from 2.7 to 42%^1, 2^. Co-occurring alcohol use hastens the time from onset of opioid use to OUD diagnosis^3^ and predicts poor treatment outcomes^4^. Alcohol is found with opioids in the blood of 14–16% of opioid overdose patients^5–7^. Oral oxycodone administered prior to oral alcohol increases ratings of drug liking and willingness to take these drugs again relative to placebo; with no effects of either drug alone on these ratings^8^. Thus, opioid-alcohol polysubstance use (PSU) is prevalent and clinically relevant, as it increases the probability of future use and worsens OUD trajectories.

Animal models of opioid-alcohol PSU are essential for the identification of neuroadaptations unique to PSU. Intravenous self-administration (IVSA) is the most widely used animal model of drug-seeking. Of the 1800 + publications on opioid IVSA (PubMed), none involve the consumption of alcohol. To begin to investigate opioid-alcohol PSU, here we assess the ability of sequential self-administration of oxycodone and alcohol to influence the motivation to seek and consume both drugs in rats. We chose oxycodone, as opposed to other opioids, because of its prevalent use and greater association with overdose deaths relative to other opioids like heroin^9, 10^. There is currently little data regarding the patterns of opioid-alcohol PSU employed (i.e. sequential, simultaneous), with the exception of reports that 16–20% of opioid users consume alcohol on the same day^11, 12^, in agreement with the prevalence of alcohol detected in the blood of opioid overdose victims. Thus, we designed a sequential model of PSU in which both drugs are consumed on the same day with overlapping effects. Alcohol access was provided after operant sessions to allow for testing of the motivation to seek/take oxycodone in the undrugged condition.

Opioid withdrawal is associated with increased motivation to seek opioids^13^. In rodents, somatic signs of withdrawal (SSW) are consistently observed following non-contingent administration and self-administration of opioids, both when “precipitated” with an opioid antagonist and spontaneously^14–17^. Non-contingent alcohol exposure reliably produces SSW in withdrawal, as does voluntary alcohol intake^18^. In addition to producing SSW, early abstinence (24–36 h) from voluntary alcohol consumption results in anxiety-like behavior in the elevated plus maze and other assays^19–21^. Interestingly, acute oxycodone is anxiolytic in male and female rats^22^. Additive or synergistic pharmacological effects of opioids and alcohol may underlie the motivation for co-consumption.

We hypothesized that the PSU condition would result in drug intake that would have additive effects on SSW and anxiety-like behavior. We also hypothesized that different patterns of neuronal activity would be observed following reinstatement of oxycodone in the PSU and oxycodone-only conditions, as was found following sequential cocaine + alcohol IVSA^23^. Specifically, we predicted that increased oxycodone-seeking in the PSU condition would be accompanied by increased c-fos expression in cell populations involved in reinstated-drug seeking: neurons that release glutamate into the striatum and central nucleus of the amygdala (CeA), namely prelimbic (PL) cortex and basolateral amygdala (BLA) neurons expressing the vesicular glutamate transporter 1 (vGlut1), and the downstream dopamine 1 receptor (D1)-expressing cells of the nucleus accumbens (NA) core and the dorsolateral striatum (dSTR)^24–30^. We hypothesized that increased reinstatement would be accompanied by decreased c-fos expression in circuits that inhibit drug-seeking, namely glutamate neurons of the infralimbic (IL) cortex^31^. To test these hypotheses, we employed a 3-phase model in which rats first established oxycodone IVSA with low-effort required to obtain drug, followed by determination of the elasticity of demand for IV oxycodone upon increases in the response requirement necessary to obtain a single infusion. During both phases, rats had access to alcohol and/or water in the home cage immediately following operant sessions. The last phase was the extinction and cue-primed reinstatement of oxycodone-seeking followed by c-fos mRNA expression analysis. We used classic inferential statistics to test the hypothesis that alcohol co-use alters oxycodone intake and seeking, anxiety-like behavior, withdrawal and reinstatement-induced c-fos expression. We also used machine-learning clustering techniques to examine the ability of sex and alcohol intake to alter the relationship between these dependent variables.

## Materials and methods

### Subjects

Sprague–Dawley rats (n = 60; half male; 8 weeks old; Charles River, Raleigh, NC) were single housed on a reversed 12-h light cycle (lights on at 7 am) in a temperature-controlled vivarium. Procedures were approved by the University of Florida IACUC, performed in accordance with these guidelines and regulations, and are reported according to ARRIVE guidelines.

### Drugs

Oxycodone HCl (Sigma Aldrich) was prepared in 0.9% physiological saline at the concentration of 0.4 mg/mL for male rats and 0.32 mg/mL for females. Alcohol (100%; Fisher Scientific) was diluted with tap water to 20% (v/v).

### Surgery

Rats were anesthetized using ketamine (87.5 mg/kg, IP) and xylazine (5 mg/kg, IP). Ketorolac (2 mg/kg, IP) was administered post-operatively and 3 days following surgery for analgesia. Catheters (SILASTIC silicon tubing, ID 0.51 mm, OD 0.94 mm, Dow Corning, Midland, MI) were implanted in the jugular vein, secured with sutures, and passed subcutaneously between the shoulder blades to exit through the skin on the back. Catheter tubing was connected to a stainless-steel cannula (Plastics One, Roanoke, VA, USA) embedded in a rubber harness (Instech, Plymouth Meeting, PA, USA) that was worn for the duration of self-administration. The antibiotic cefazolin (100 mg/kg) was administered IV (0.1 mL) for 3 days post-surgery. Catheters were flushed with heparin (100 IU/mL; 0.1 mL) before and after each self-administration. Catheter patency was tested periodically with methohexital sodium (10 mg/mL; Eli Lilly, Indianapolis, IN, USA), which results in a temporary loss of muscle tone.

### Self-administration, demand analyses, extinction and reinstatement

**Figure 1:**
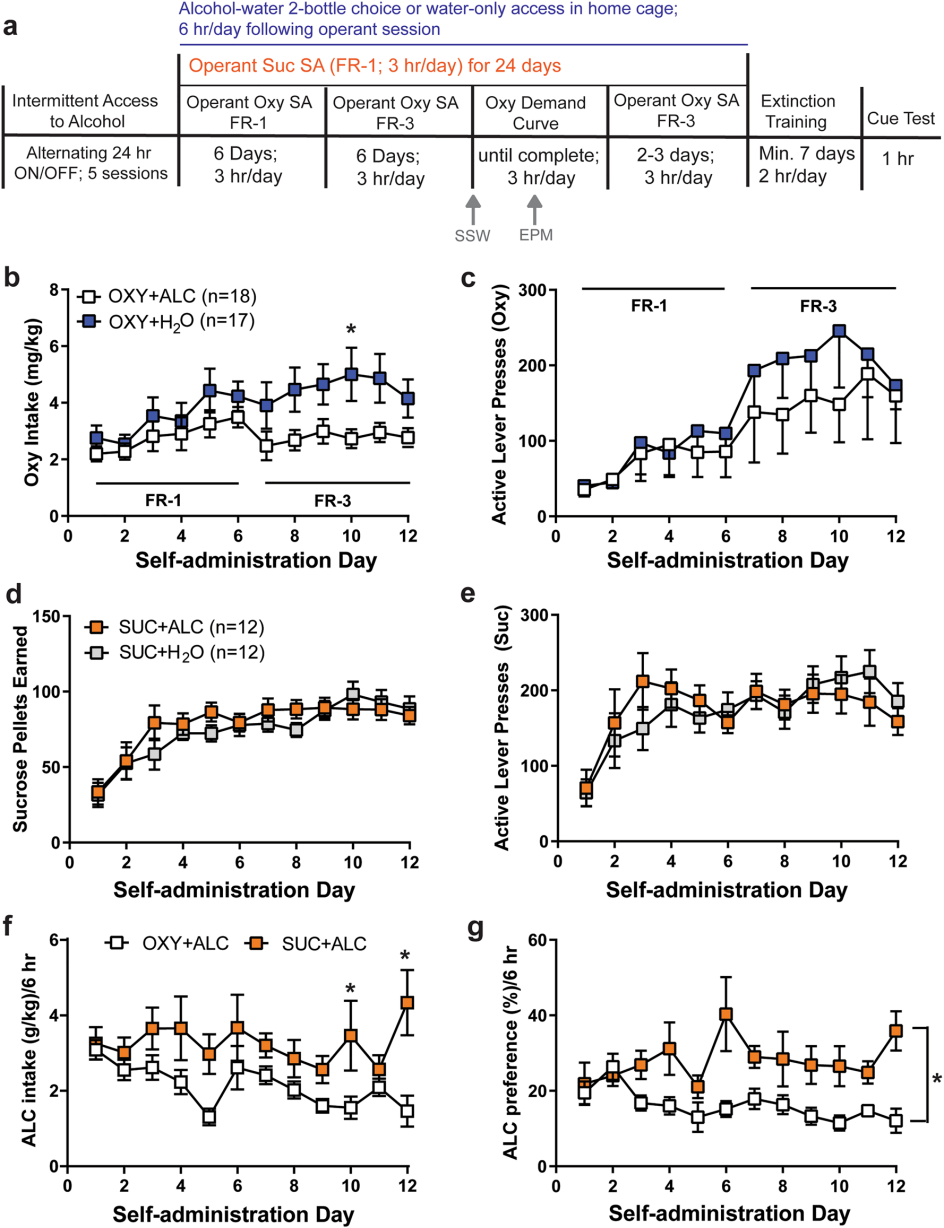
Alcohol and oxycodone influence the intake of one another. (**a**) Timeline for the experiment. All rats experienced either 2-bottle choice or water access in the homecage. Rats self-administered either oxycodone (OXY) or sucrose (SUC) in the operant chamber for 12 days; OXY self-administering rats then underwent demand curve analyses, while the sucrose self-administering rats did not. Alcohol (ALC) and/or water (H_2_O) access was provided daily during this time, immediately following the operant session. Rats underwent extinction training in the absence of alcohol access, followed by a cue-primed reinstatement test. There were no effects of sex on oxycodone, sucrose and alcohol self-administration and thus males and females are graphed together here. (**b**) For oxycodone intake, there was a Liquid × Time interaction [F_(11, 363)_ = 2.352, p = 0.0082], and the Oxy + H_2_O condition displayed greater oxycodone intake than the Oxy + ALC condition on Day 10. (**c**) Access to alcohol had no effect on active lever presses for oxycodone, which increased over time [main effect of Time: F_(11, 363)_ = 9.997, p < 0.0001]. (**d**) Sucrose intake increased over time [F_(11,242)_ = 18.130, p < 0.0001] as did active lever presses (**e**) for sucrose SA [F_(11,242)_ = 8.435, p < 0.0001]. (**f**) There was a Time × Liquid interaction [F_(11, 297)_ = 1.943, p < 0.0351] for alcohol intake; with sucrose self-administering rats consuming more alcohol than oxycodone self-administering rats on Days 10 and 12. (**g**) For alcohol preference, there was a significant effect of Reinforcer [F_(1, 25)_ = 14.196, p = 0.0081], with rats undergoing oxycodone self-administration showing a reduced preference for alcohol relative to sucrose rats. N = 9 female OXY + ALC, n = 9 male OXY + ALC, n = 9 female OXY + H_2_O; n = 8 male OXY + H_2_O, N = 6 female SUC + ALC, n = 6 male SUC + ALC, n = 6 female SUC + H_2_O; n = 6 male SUC + H_2_O. *p < 0.05 vs. OXY + ALC.

One week after arrival, rats that would later have access to alcohol (alcohol) following operant sessions were exposed to intermittent access to alcohol (IAA). Rats were provided 2-bottle choice for unsweetened alcohol in the home cage in five 24-h sessions alternating with 24 h periods without alcohol access (see timeline in Fig. 1a), as previously done in a sequential model of cocaine + alcohol^23, 32^. Rats were then implanted with jugular catheters and permitted 6 days to recover. Thirty-six rats underwent oxycodone IVSA (0.1 mg/kg/infusion^33^) for 3 h/day for 6 days on an FR-1 schedule of reinforcement, followed by 6 days on an FR-3 schedule. Self-administration took place in 2-lever operant chambers (Med Associates, Inc). Presses on the active lever delivered IV oxycodone and drug-associated cues (stimulus light and 2900 Hz tone). There was a 20-s time out following reinforcer delivery, during which time presses were recorded but reinforced. Presses on the inactive lever had no programmed consequences. Twenty-four rats (half male) self-administered 45 mg sucrose pellets (BioServ) instead of oxycodone in the same manner. Immediately following the SA session, rats were placed into the home cage for 6 h access to 2-bottle choice or water only (n = 18 oxycodone and n = 12 sucrose in each condition (alcohol/water).

After 12 days of IVSA, demand for oxycodone was assessed. Demand curve protocols consider consumption of a reinforcer at increasing price (i.e., lever presses) in order to determine flexibility, or elasticity, of reinforcer-seeking. Demand curve sessions were identical to oxycodone IVSA training sessions, except that the FR requirement to attain an infusion of oxycodone was increased by quarter- and then eighth-log units (e.g., FR-6, FR-10, FR-13) every second day. Access to alcohol and/or water continued as originally assigned. Sucrose rats did not undergo demand curve procedures, but self-administered sucrose on an FR-1 for 24 sessions. After completion of the demand procedure (earning 0 infusions on both days of a FR), rats re-established oxycodone IVSA on an FR-3 for 2–3 days until infusions were within 25% of the training average, followed by instrumental extinction training for a minimum of 7 days and until meeting criterion (< 25% active lever presses during last day of IVSA). Sucrose rats underwent extinction training to the same criteria. Rats that met criteria by Day 16 were tested for cue primed reinstatement in a 1-h test, during which the active lever yielded reinforcer-associated cues. Rats were killed immediately via rapid decapitation without anesthesia, brains flash frozen and stored at – 80 °C. This timing was chosen as it captures the peak of c-fos mRNA expression^34^. See Fig. 1a for timeline; the same cohort of rats was used for the entire study.

### Assessment of spontaneous withdrawal and anxiety-like behavior

After completing 12 days of self-administration, rats were assessed for spontaneous somatic signs of withdrawal (SSW) 20–22 h after the last self-administration session (Fig. 1a). Rats were placed into a clear plexiglass chamber (16″ W × 16″ D × 15″ H) positioned under a camera for a 20-min assessment. Videos were later rated by a researcher blind to condition for behaviors commonly observed in withdrawal from opioids^17, 35^: wet dog shakes, biting, grooming, jumping, and scratching. The number of occurrences of these behaviors were summed to generate a global withdrawal score.

All rats completed 18 days of self-administration (FR-18). Anxiety-like behavior was assessed at 20–22 h withdrawal following the last FR-18 session. Rats were tested on the elevated plus maze (EPM) using an apparatus from Med Associates (St. Albans, VT, USA). The black plexiglass EPM had four arms (50 cm length × 10 cm width) raised 72 cm from the floor. Two open arms (1.5 cm high walls) and two closed arms (50 cm high walls) were joined by a center square platform (10 cm × 10 cm). The center platform was illuminated at 50 lx. Rats were individually placed on the center platform and given 5 min to explore the maze. Time spent in the open arms (OA), closed (CA), and number of OA and CA entries were measured using EthoVision XT 14 software (Noldus Information Technology, Leesburg, VA). Anxiety-like behavior and SSW were assessed at the 20–22 h withdrawal from oxycodone/sucrose to correspond with the time at which rats would be placed into the operant chamber for the subsequent self-administration session in order to uncover potential roles for these side-effects in motivating self-administration.

### Fluorescent in situ hybridization (FISH) and Imaging for the quantification of c-fos mRNA expression

Coronal sections (14 µM) were obtained on a Leica CM1950 cryostat and underwent FISH using probes for c-fos and D1 or vGlut1. Regions of interest included the PL and IL cortices, NA core (NAc) and shell (NAs), dSTR, BLA, and the central nucleus of the amygdala (CeA). Fluorescent in situ hybridization was performed using the RNAscope Multiplex Fluorescent Reagent Kit (Advanced Cell Diagnostics; Newark, CA, USA) according to the manufacturer’s instructions with some modifications (Shallcross et al., 2021). Slide-mounted frozen tissue was fixed for 15 min in 4% paraformaldehyde (PFA; 4 °C, pH 7.45), and then dehydrated in an ethanol gradient (50%, 70%, 100%). Following protease digestion, slides were twice washed with PBS and hybridized with probes for c-fos in combination with either D1 (striatum) or vGluT1 (cortex and amygdala) for 2 h at 40 °C. The following probes (Advanced Cell Diagnostics) were used: c-fos (FOS, Cat #403591), D1 (Drd1a, Cat. # 317031), and vGlut1 (SLC17A7; Cat. #317001). In the PL, IL, and BLA c-fos and vGlut1 probes were used, as glutamate neurons in these regions projecting to the striatum have been shown to regulate the reinstatement of drug-seeking^36–38^. In the dSTR, NAc and NAs, c-fos and D1 probes were used, as D1-containing neurons have been shown to drive reinstated drug-seeking^39^. Antifadant mounting media containing DAPI counterstain was used.

### Image acquisition and quantification

A Leica DM6B microscope equipped with a sCMOS K5 camera and LASX software (v.3.7.423463) was used to acquire images using a 40 × objective. In each region, 10 µm z-stacks were taken with 1 µm steps. Two sections were imaged from each brain region/rat by a researcher blind to condition. Colocalization and quantification of mRNA transcripts were done with CellProfiler (v. 4.0.7; http://cellprofiler.org/). mRNA expression was quantified within a 10-pixel region surrounding nuclei. The dependent measures were the number of c-fos + cells and the number of c-fos/vGluT1 or c-fos/D1 doubled labeled cells.

### Statistical analyses

Data were analyzed using SPSS (v.28, IBM) for 3-way ANOVAs and GraphPad Prism (v.9.4.1, GraphPad Software) for all other parametric tests and demand curve analyses. R (v. 4.2.2) was used to conduct the multi-dimensional scaling (MDS) analysis^40^. 3-way ANOVAs compared self-administration variables (Sex × Liquid × Time), alcohol intake/preference (Sex × Reinforcer × Time), and EPM and SSW dependent measures (Sex × Liquid × Reinforcer). Alcohol preference was computed by dividing the amount of alcohol consumed by the total amount of liquid consumed. Reinstatement of oxycodone-seeking was defined as a significant increase in active lever presses during the cue test compared to the last day of extinction; this comparison was made using paired-samples t-tests with the a priori hypothesis that all groups would reinstate. Significant interactions were followed by Sidak’s post-hocs. The Grubbs test was used to screen data for the presence of outlier values.

Economic demand for oxycodone was assessed by plotting drug consumption (mean infusions averaged over the 2 days of each FR) as a function of FR and fitting the equation log*Q* = log(*Q*_0_) + k(e^−α*P*^ − 1)^41^. *Q* is the number of infusions at each “price” (*P*), or FR requirement. *K* is a fixed scaling parameter representing the range of the dependent variable in logarithmic units, which for this data set was 4.031. Dependent measures include Q_0,_ α, essential value, and P_max_. Q_0_ is the y-intercept, and estimates drug consumption at an FR of 0 such that a larger value indicates increased consumption of the drug when it is “free”. Alpha (*α*) is an index of elasticity of demand, which reflects the rate of change in consumption as a function of response requirement increases; α diminishes when the seeking of the reinforcer is less flexible. Here we computed log α to meet normality assumptions for subsequent analyses. Essential value, or α transformed, is calculated with the formula 1/(100αk^1.5^), and is the rate of change of the elasticity of demand, such that lower values indicate greater change in demand elasticity^41^. P_max_ is calculated with the formula m/(Q_0_αk^1.5) where m = 0.084 k + 0.65. Reinforcer intake at prices (FR values) lower than P_max_ is relatively stable, or “inelastic”, while intake at FR values higher than P_max_ diminishes rapidly upon response requirement increases, and is “elastic”^41, 42^. The extra sum-of-squares F-test was used as a nonparametric test of the null hypothesis that *α* did not differ between groups and 2-way (Liquid × Sex) ANOVAs compared (log) *α*, *Q*_0,_ essential value, and P_max_ between conditions^42^.

mRNA expression was averaged between two images/region for each rat. For the oxycodone groups, a 3-way Sex × Liquid × Test ANOVA was conducted to identify regions which responded to the cue test. Expression in tested and untested rats was then analyzed separately with 2-way Sex × Liquid ANOVAs. For sucrose rats, 2-way Sex × Liquid ANOVAs compared c-fos expression between conditions. These analyses were conducted separately for oxycodone and sucrose rats because the goal was to test the hypothesis that alcohol alters reinstatement-induced c-fos expression in oxycodone rats while having no effect in sucrose rats. Pearson’s correlations assessed the relationship between select dependent variables. Significant interactions were followed by Sidak’s post-hoc tests, correcting for multiple comparisons.

To examine the ability of Sex and Liquid to change the relationship between dependent variables, multidimensional scaling (MDS) analysis was used. MDS uses statistical approaches to graphically represent the similarity/dissimilarity between variables. Similarity was based on Pearsons correlations (computed via the R package *cor*), that were calculated within each condition (oxycodone + alcohol male and female; oxycodone + water male and female; sucrose + alcohol and sucrose + water). Sucrose conditions were not examined by Sex due to the low n for many variables. Uncorrected p-values were calculated using *rcorr$p*. All correlation coefficients were converted to absolute values (i.e., abs(r)), which were used to produce a distance matrix based on 1-abs(r) which was then subjected to MDS analysis. Subsequently, groups of correlated behaviors were identified using K-means clustering via the R function *kmeans* which applies a machine learning clustering algorithm to identify clusters of correlated variables. *Nbclust* was used to identify the optimal number of clusters and instructed to identify the optimum a range of 2–7 clusters. The main variables of interest were oxycodone and alcohol intake, drug-seeking during the cue test, withdrawal score and anxiety-like behavior, and c-fos expression. In cases where variables were inherently related and inferential statistics found similar group differences (e.g., time spent in the OA and time in CA; oxycodone intake and infusions; Essential Value and log α), we selected only one representative variable. Three week totals for alcohol and oxycodone intake were used in the analysis as they best represent each drug’s impact on the brain. The inclusion of 2-week totals did not change cluster composition. Day 1 of extinction training was included as it is an index of both reinforcement learning and seeking. SSW could not be incorporated into the analysis for sucrose rats, since the three rats without SSW ratings were some of those that were tested for reinstatement. For sucrose rats, based on smaller animal numbers, analyses were not separated by sex. Because there was no effect of sex on dependent measures for the sucrose self-administering rats (with the exception of c-fos expression in the NA core), sex would likely have less of an effect on MDS outcomes in sucrose rats.

## Results

At the onset, oxycodone + alcohol and oxycodone + water conditions comprised 18 rats, half of which were male. One male oxycodone + water died during IVSA; 5 rats completed IVSA but died/lost patency during the demand curve (2 female and 1 male oxycodone + alcohol, 1 male and 1 female oxycodone + water). Two rats died during extinction (1 male oxycodone + alcohol, 1 female oxycodone + water). All sucrose rats completed self-administration. Data from one female sucrose + alcohol rat was eliminated from analysis as alcohol intake was found to be an outlier with the Grubbs test. Assessment of SSW was not completed for 2 additional female sucrose rats due to experimenter error. BLA vGluT1 signal was undetectable for many oxycodone rats and so only c-fos^+^ cells were counted in this region.

### Oxycodone, alcohol and sucrose self-administration

There were no 2- or 3-way interactions with the factor of Sex for any self-administration variable and thus results are depicted in Fig. 1 without considering this factor. See Supplementary Fig. S1 for depictions of data accounting for sex. Access to alcohol reduced oxycodone intake (Fig. 1b) and infusions earned (Supplementary Fig. S1), with no effect on active or inactive lever presses during oxycodone self-administration (Fig. 1c), sucrose intake (Fig. 1d), or active or inactive lever presses during sucrose self-administration (Fig. 1e). Rats undergoing oxycodone IVSA consumed less alcohol (Fig. 1f) and displayed a reduced preference for alcohol (Fig. 1g) relative to sucrose rats. The sucrose + alcohol group consumed a greater total amount of alcohol during the first 12 days of self-administration than did the oxycodone + alcohol group [t_(27)_ = 3.132, p = 0.0041; mean oxycodone + alcohol: 25.55 ± 2.185; mean sucrose + alcohol: 39.21 ± 4.313 g/kg.].

### Somatic signs of withdrawal and anxiety-like behavior

**Figure 2:**
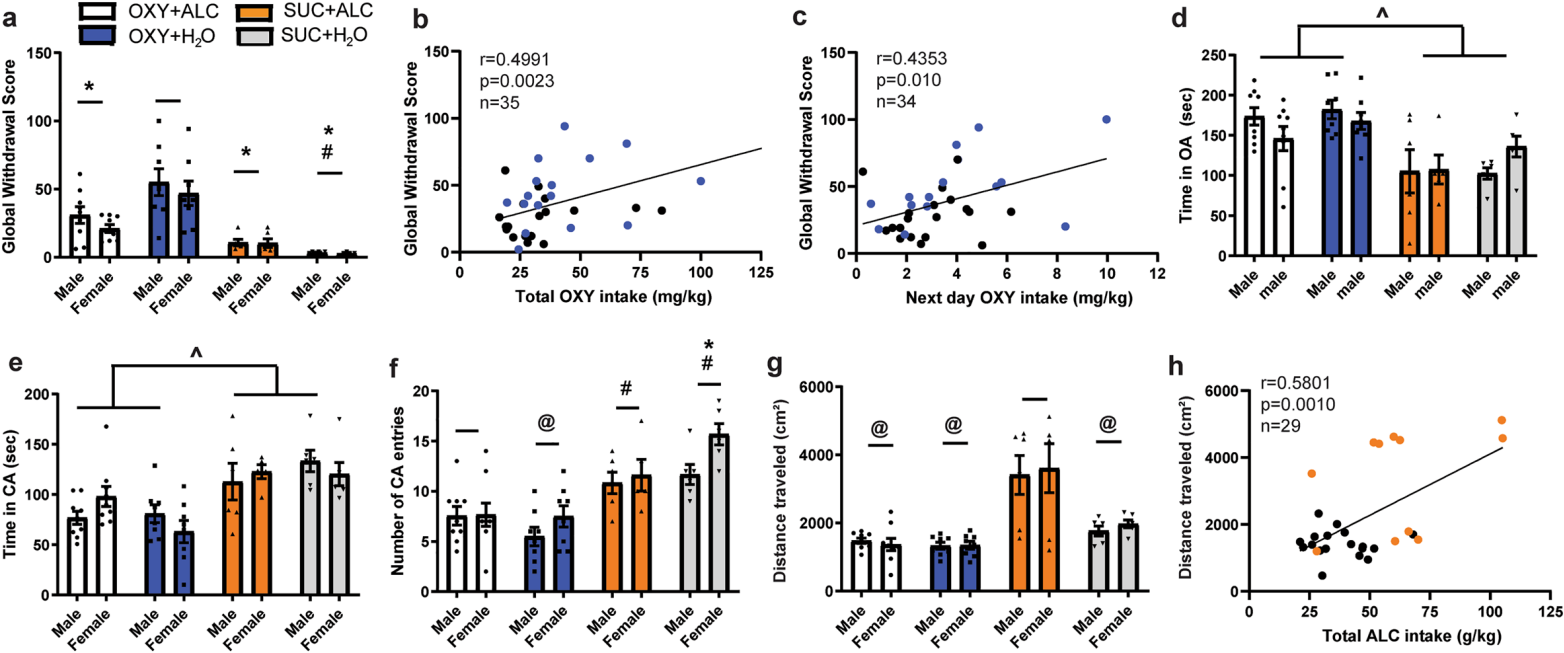
At the time at which rats begin self-administration sessions, oxycodone self-administering rats display greater somatic signs of withdrawal and less anxiety. (**a**) There were no sex differences in the number of somatic signs of withdrawal exhibited 24 h after the last operant session. There was a Reinforcer × Liquid interaction [F_(1,47)_ = 8.541, p = 0.005], with OXY + H_2_O rats displaying greater withdrawal signs than all other groups and OXY + ALC rats displaying withdrawal signs greater than the SUC + H_2_O group. (**b**) The global withdrawal score positively correlated with the total amount of oxycodone intake in the 12 days prior to assessment [r(35) = 0.4991, p = 0.0023] but not with the total amount of alcohol intake (not shown). The global withdrawal score positively correlated with the amount of oxycodone intake on the day immediately following SSW assessment [r(33) = 0.4425, p = 0.0099]. For both (**b**,**c**) blue dots represent Oxy + H_2_O rats and black dots are OXY + ALC rats. (**d**) A main effect of Reinforcer [F_(1,49)_ = 27.227, p < 0.001] was found for time spent in the open arms (OA) of the EPM. Rats that consumed oxycodone spent more time in the OA than sucrose-consuming rats, indicating reduced anxiety. (**e**) There was a main effect of Reinforcer for time spent in the closed arms [CA; F_(1,49)_ = 30.609, p < 0.0001] with both sucrose groups spending more time in the CA than the oxycodone groups. (**f**) There was a Liquid × Reinforcer interaction for the number of entries into the CA [F_(1,49)_ = 5.135, p < 0.028], with both sucrose groups exhibiting more entries than oxycodone groups. (**g**) There was a Liquid × Reinforcer interaction for locomotion in the EPM [F_(1,49)_ = 11.922, p = 0.001], with the SUC + ALC group displaying greater locomotion than all other groups. (**h**) There was a positive correlation between locomotor activity in the EPM and total alcohol consumed. Orange dots represent SUC + ALC rats and black dots represent OXY + ALC rats. *p < 0.05 vs. OXY + H_2_O; ^#^p < 0.05 vs. OXY + ALC; ^^^p < 0.05 comparing OXY to SUC; ^@^p < 0.05 vs. SUC + ALC.

Oxycodone + water rats displayed greater withdrawal signs than all other groups and oxycodone + alcohol rats displayed withdrawal signs greater than the sucrose + water group (Fig. 2a). The global withdrawal score was positively correlated with the total amount of oxycodone self-administered in the 12 days prior to assessment (Fig. 2b), as well as future oxycodone intake on Day 13, 24 h following SSW assessment (Fig. 2c), but not alcohol intake (not shown). See Supplemental Fig. S2 for individual withdrawal signs, which followed a similar pattern as the global score with the exception of wet dog shakes which were greater in male oxycodone self-administering rats. Jumps were only observed in 2 rats and are not reported here.

Oxycodone + alcohol and oxycodone + water rats spent more time in the open arms than sucrose rats, and less time in the closed arms (Fig. 2d,e). Sucrose self-administering rats exhibited more closed arm entries than oxycodone rats (Fig. 2f). Open arm entries did not differ between groups (not shown). The sucrose + alcohol group displayed greater locomotion in the EPM than all other groups (Fig. 2g). Total alcohol (and not oxycodone) intake was positively correlated with distance traveled in the EPM (Fig. 2h), indicating withdrawal-induced hyperlocomotion.

### Economic demand for intravenous oxycodone

**Figure 3:**
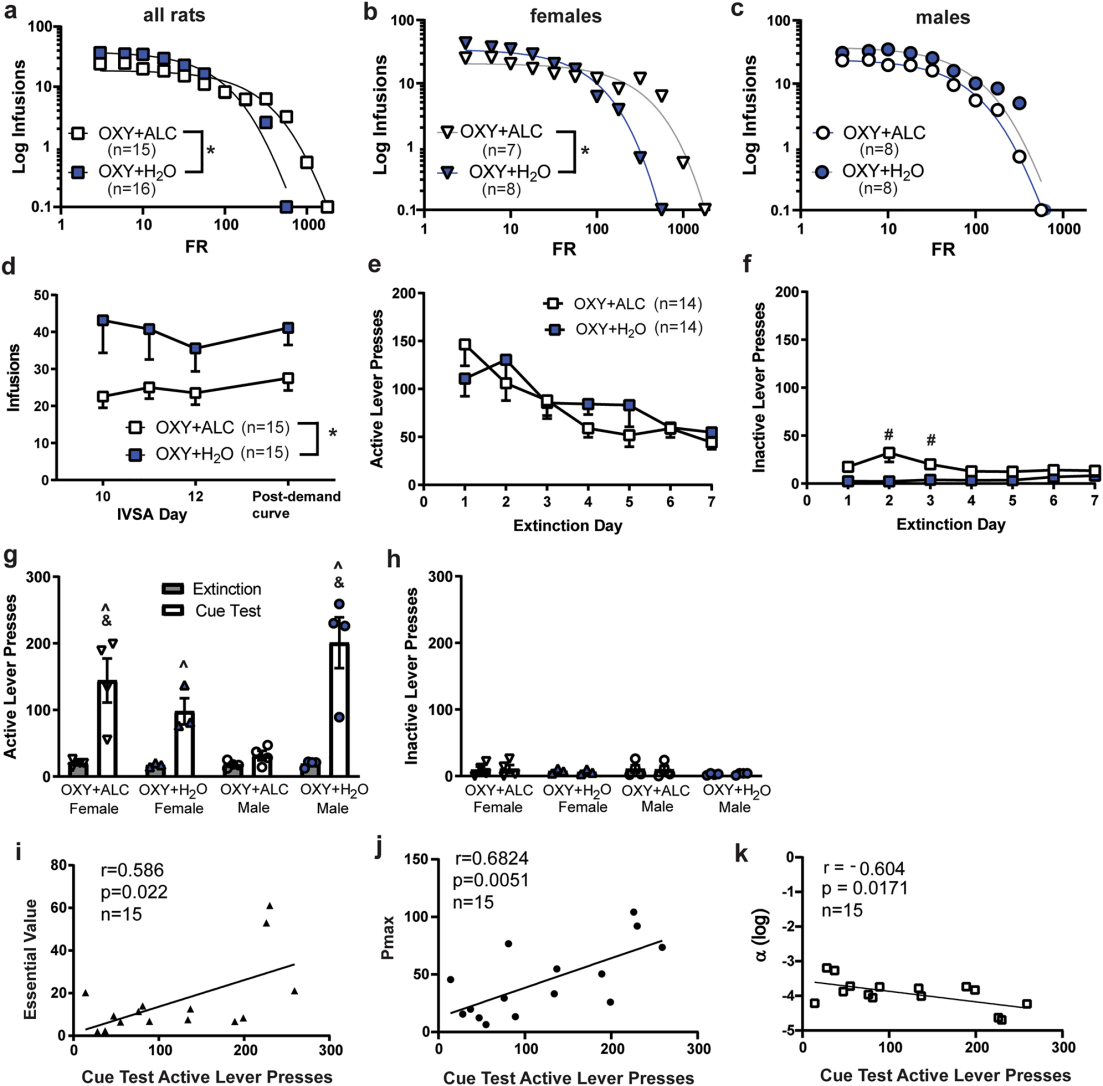
Demand for intravenous oxycodone is increased by co-consumption of alcohol in female but not male rats. (**a**) Demand curve analyses comparing all rats found that rats that consumed both oxycodone and alcohol displayed less elastic demand for oxycodone [F_(1, 23)_ = 10.26, p < 0.0001]. (**b**) However, considering sex in these analyses reveal that this effect is driven by females [F_(1, 18)_ = 34.81, p < 0.0001] with no group differences evident in male rats (**c**). (**d**) Oxycodone self-administration readily resumed on an FR-3 schedule after the demand curve. There was an effect of Liquid [F_(1, 27)_ = 6.716, p = 0.0152] on infusions during this phase, with alcohol-consuming rats attaining less oxycodone before and after the demand curve. (**e**) Extinction of oxycodone seeking did not differ by sex; both groups extinguished responding similarly evidenced by a main effect of Time [F_(6, 156)_ = 10.722, p = 0.005]. (**f**) There was a Liquid × Time interaction [F_(6, 156)_ = 4.817, p = 0.045] for inactive lever pressing during extinction training; pressing was higher for the OXY + ALC group on days 2 and 3. (**g**) Alcohol co-use increases cue-primed reinstatement of oxycodone-seeking in female rats and reduces it in male rats. A significant Sex × Liquid × Time interaction was found for active lever pressing during the last day of extinction and the cue primed reinstatement test [F_(1,11)_ = 13.217, p = 0.004]. Post-hoc tests found that the female OXY + ALC and female and male OXY + H_2_O groups reinstated seeking (significant difference between extinction and test), while the male OXY + ALC rats did not. The OXY + H_2_O males displayed greater lever presses during the test compared to the male OXY + ALC rats. (**h**) Inactive lever pressing during the test was low and did not differ between groups. (**i**) Essential Value (α transformed) computed from the oxycodone demand curve analyses was positively correlated with active lever presses during the cue test. (**j**) Active lever pressing during the cue test was positively correlated with P_max_ derived from oxycodone demand curve analyses. (**k**) Demand elasticity (log α) was negatively correlated with active lever pressing during the cue test. ^#^p < 0.05 vs. OXY + H_2_O; ^^^p < 0.05 vs. extinction; ^&^p < 0.05 vs. male OXY + ALC. *Effect of Liquid p < 0.05.

Demand curve analyses revealed a significant reduction in elasticity of demand (α) for oxycodone in the oxycodone + alcohol group (Fig. 3a). However, when considering sex, only female oxycodone + alcohol rats displayed less elasticity of demand relative to female oxycodone + water rats (Fig. 3b) with no differences in males (Fig. 3c). From individual demand curves, Q_0_, essential value, and P_max_ can be calculated. No group differences were found for these variables when compared between groups (Supplementary Table S1).

### Reinstatement of oxycodone-seeking and associated c-fos mRNA expression

Rats readily re-established responding for oxycodone on an FR-3 schedule (Fig. 3d; Supplementary Fig. S3). All rats completed at least 7 days of extinction training; during these 7 days, there were no 3- or 2-way interactions on active lever presses (Fig. 3e). The oxycodone + alcohol condition displayed increased inactive lever presses on Day 2 and 3 of extinction (Fig. 3f), which declined over training. Only 4 of 7 rats from each condition met extinction criteria by Day 16 and were tested for reinstatement. Male oxycodone + water and female oxycodone + alcohol rats displayed greater reinstatement active lever presses than male oxycodone + alcohol rats (Fig. 3g). When comparing lever pressing during the test to those during the last two days of extinction training with post-hoc tests, only the male oxycodone + water and female oxycodone + alcohol groups increased responding during the test, i.e. reinstated responding (p’s < 0.01). A paired samples t-test found that the oxycodone + alcohol males only displayed a trend towards an increase in responding from extinction to test (p = 0.08). There were no effects on inactive lever pressing during extinction and test (Fig. 3h). Reinstatement active lever pressing was correlated with measures that are indexes of flexibility of demand under the conditions of increasing costs: reinforcer efficacy (Fig. 3i), P_max_ (Fig. 3j), and (log) α (Fig. 3k). These results indicate a strong relationship between oxycodone-seeking during economic demand procedures and reinstatement tests.

**Figure 4:**
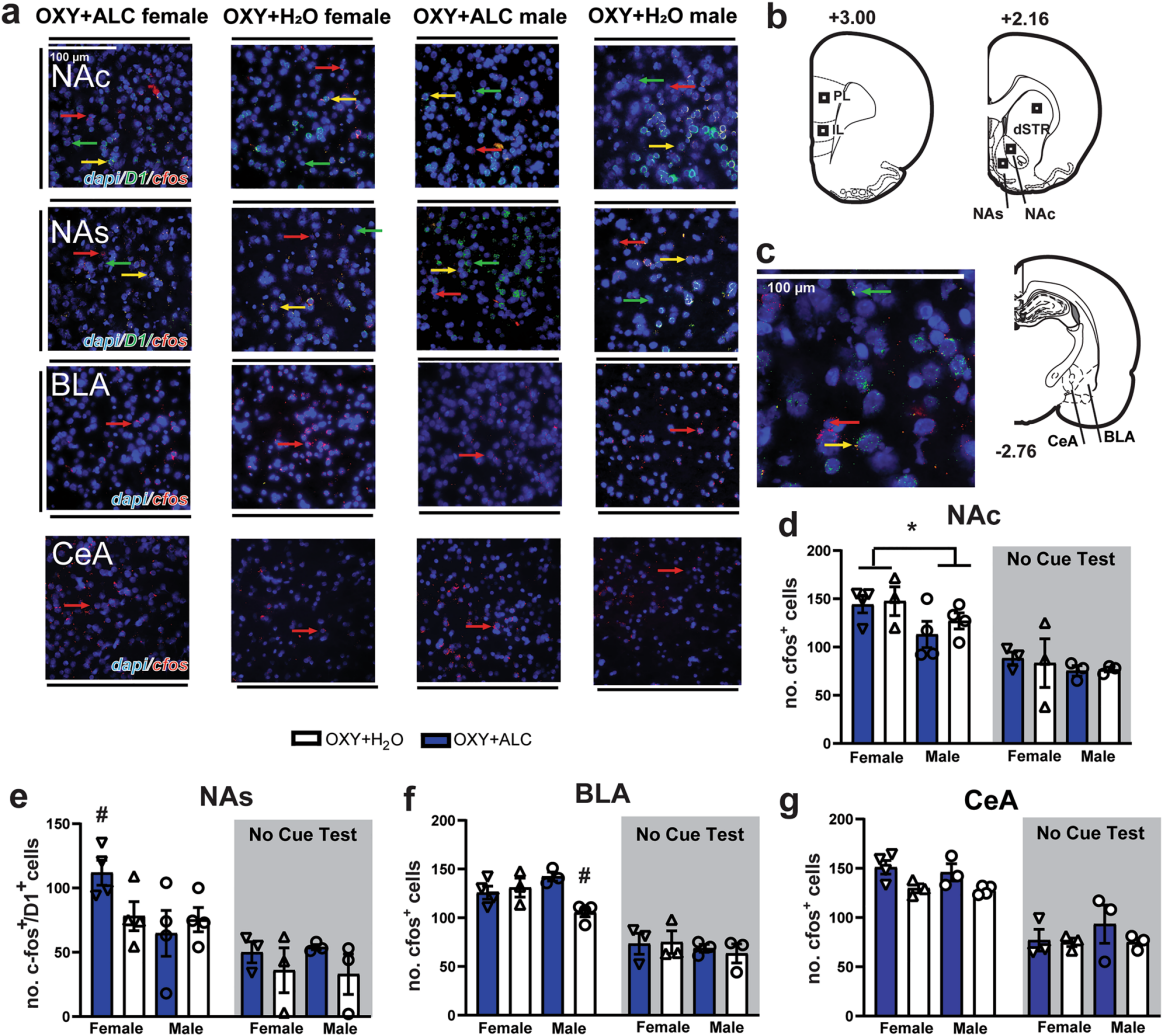
C-fos expression was induced by the cue test and was influenced by alcohol and sex in select brain regions and cell populations. (**a**) Representative images (40x) from regions and cell populations that showed increased c-fos expression during the test relative to the no-test condition in a manner influenced by a history of alcohol consumption and/or sex. (**b**) Sites of analysis in the prelimbic (PL) and infralimbic (IL) cortices, nucleus accumbens (NA) shell (NAs), core (NAc), dorsal striatum (dSTR), and the basolateral nucleus (BLA) and central nucleus (CeA) of the amygdala. (**c**) Representative image illustrating D1/c-fos co-expression in the NAc. (**d**) In the NAc, there was a main effect of Sex [F_(1,11)_ = 8.816, p = 0.0128], with females displaying greater number of c-fos^+^ cells. (**e**) In the NAs, there was a significant interaction for the number of c-fos^+^/D1^+^ cells [F_(1,11)_ = 6.117, p < 0.031], with females displaying more c-fos^+^/D1^+^ cells than males in the ALC condition. (**f**) In the BLA, there was a Sex × Liquid interaction for the number of c-fos^+^ cells [F_(1,11)_ = 10.29, p < 0.009], with post-hoc tests finding that male OXY + ALC rats displayed a greater number than male OXY + H_2_O rats. There were no group differences in the “no cue test” condition in the BLA, or the CeA. (**g**) In the CeA, upon a reinstatement test, alcohol-consuming rats displayed greater number of c-fos^+^ cells [main effect of Liquid: F_(1,10)_ = 11.04, p = 0.0077]. ^&^Effect of Sex p < 0.05; *Effect of Liquid p < 0.05; ^#^p < 0.05 vs. OXY + ALC males.

Sites of imaging and representative images are shown in Fig. 4a–c. It was first determined which brain regions were active during the cue-test by comparing expression in rats that underwent reinstatement tests with those that did not with 3-way (Sex × Liquid × Test) ANOVAs. No 3-way interactions were detected (see Supplementary Table S2). For the number of double-labeled cells (c-fos^+^/vGlut^+^ or c-fos^+^/D1^+^), there was a main effect of Test (i.e., increased c-fos expression in the tested rats) for the IL, NAc, NAs, and dSTR, but not PL (Supplementary Fig. S3). There was an effect of Test on number of c-fos^+^ cells only in the IL, BLA, CeA, and NAc, indicating that most cells activated by the test are D1-expressing in the NAs and dSTR.

Next, Sex × Liquid ANOVAs were conducted for test and no-test conditions separately, finding no significant effects on c-fos expression in any region for the no-test condition. Thus, in the absence of a test, c-fos expression does not differ based on sex or alcohol consumption. In the Test condition, females displayed a greater number of c-fos^+^ cells in the NAc (Fig. 4d), an effect that occurred at the trend level for c-fos^+^/D1^+^ cells (p = 0.07; Supplementary Fig. S3). In the NAs, females displayed more c-fos^+^/D1^+^ cells than males in the alcohol condition (Fig. 4e), with no effects on the number of c-fos^+^ cells (Supplementary Fig. S3). There were no significant effects in the dSTR (Supplementary Fig. S3). For the BLA (Fig. 4f), male oxy + alcohol rats displayed greater activation than male oxy + water rats, and in the CeA, alcohol-consuming rats displayed greater c-fos expression (Fig. 4g). The number of c-fos^+^ cells in the BLA negatively correlated with active lever pressing during the cue test [r(14) = − 0.6178, p = 0.0123]. There were no effects in the PL and IL (Supplementary Fig. S3).

### Reinstatement of sucrose-seeking and associated c-fos mRNA expression

**Figure 5:**
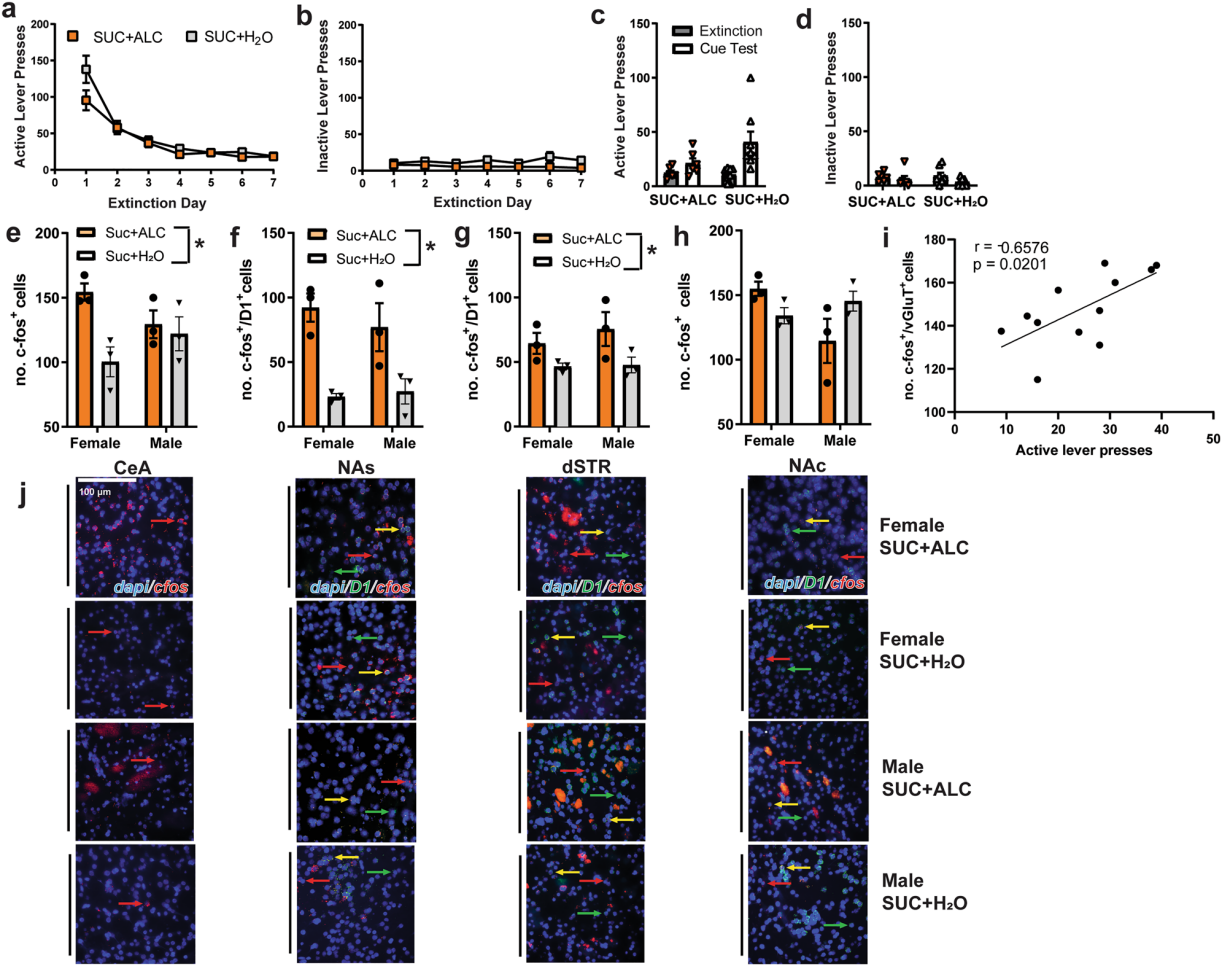
Alcohol intake does not influence lever pressing during extinction and cued reinstatement of sucrose-seeking but increases c-fos expression following a cue test. (**a**). Active lever pressing decreased during extinction training and did not differ between conditions [main effect of Time: F_(6, 114)_ = 99.904, p < 0.0001]. (**b**) Inactive lever pressing did not differ between water and alcohol-consuming groups. (**c**) There was a significant effect of Time [F_(1,11)_ = 11.604; p = 0.0018], but no interactions when comparing lever pressing on the last day of extinction to pressing during the cue test. Males and females are graphed together due to the lack of any effects of Sex on this behavior. (**d**) Inactive lever pressing decreased slightly during the test [main effect of Time: F_(1,11)_ = 6.972, p = 0.0332] and did not differ by group. Despite no effects of alcohol on lever pressing during the cue test, there was a lasting effect of alcohol-consumption on c-fos expression in the (**e**) CeA [F_(1, 8)_ = 8.103, p = 0.0216], and more c-fos^+^/D1^+^ cells in the (**f**) NAs [F_(1, 8)_ = 24.85, p = 0.011], and (**g**) dSTR [F_(1, 8)_ = 7.394, p = 0.0263] with alcohol-consuming rats displaying increased c-fos expression. (**h**) There was a Sex × Liquid interaction on c-fos expression in the NAc, with post-hoc tests finding no significant differences between groups [F_(1,8)_ = 6.327, p = 0.0361]. (**i**) Only PL c-fos expression correlated with active lever pressing during the test [r(12) = 0.6576, p = 0.0201]. (**j**) Representative images. *Effect of Liquid p < 0.05; ^&^Effect of Sex p < 0.05. Red arrows show c-fos mRNA, green arrows indicate D1 mRNA expression and yellow indicates cells where both mRNA are present. *Effect of Liquid p < 0.05; ^&^Effect of Sex p < 0.05.

There were no effects of Sex on extinction or reinstatement of sucrose seeking (Supplementary Fig. S4). Active lever pressing decreased during extinction training (Fig. 5a); inactive lever presses were low and did not differ between groups (Fig. 5b). To be consistent with methods for the oxycodone self-administering rats, 3–4 rats/sex/condition were tested for sucrose reinstatement. When comparing active lever presses from extinction to the test, there was only a main effect of Time, indicating that rats reinstated sucrose-seeking without effects of sex or alcohol on this behavior (Fig. 5c). Inactive lever pressing was not influenced by alcohol, but decreased during the test (Fig. 5d).

Only 2 sucrose rats/condition were not tested for reinstatement and thus this tissue was not hybridized. Despite no effects of alcohol on lever pressing during the cue test, a history of alcohol increased the number of c-fos^+^ cells in the CeA (Fig. 5e) and the number of c-fos^+^/D1^+^ cells in the NAs (Fig. 5f) and dSTR (Fig. 5g), with alcohol-consuming rats displaying increased c-fos expression. The number of c-fos^+^ cells in the NAc were altered by alcohol in a sex-dependent manner, with post-hoc tests finding no significant differences between groups (Fig. 5h). The only region in which active lever pressing during the sucrose test was correlated with c-fos expression was the PL (Fig. 5i). Representative images are shown in Fig. 5j. There were no effects on the number of c-fos^+^ cells in glutamatergic cells of the PL, IL, and BLA or in D1^+^ cells of the NAc (Supplementary Fig. S4).

### Multidimensional scaling

**Figure 6:**
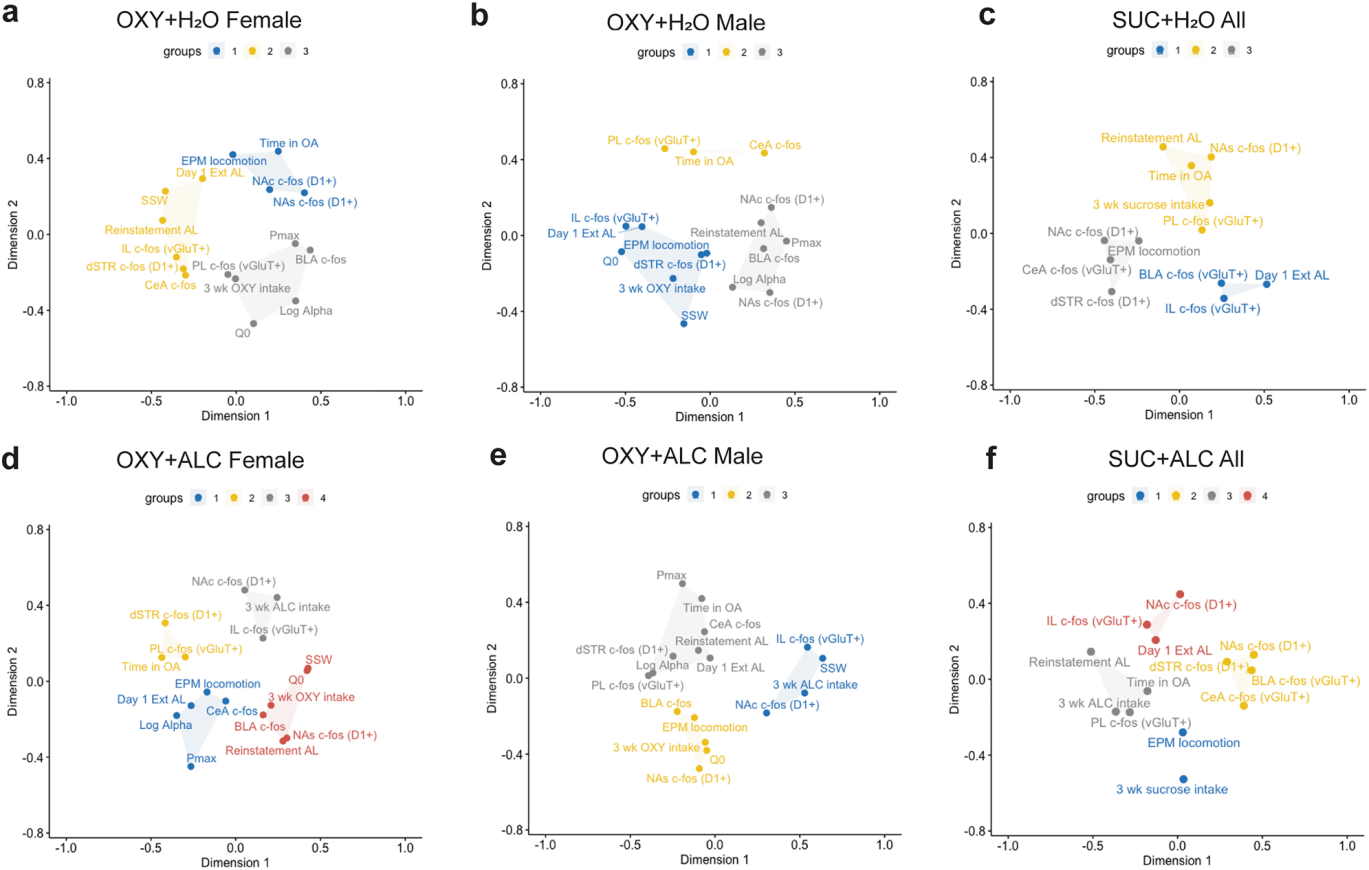
Multidimensional Scaling analysis of key dependent variables. Alcohol altered cluster composition in female (**a**,**d**) and male rats (**b**,**e**) that self-administered oxycodone, as well as that of sucrose self-administering rats (**c**,**f**). For sucrose and female oxycodone rats, the addition of alcohol consumption to the analysis yielded a fourth cluster. For female oxycodone self-administering rats, there are more differences than similarities between alcohol- and water-consuming groups, with the only relationships remaining being that 3 week total oxycodone intake clusters with Q_0_ and BLA c-fos and that Day 1 of extinction active lever presses clusters with CeA c-fos expression. For males, there are slightly more similarities in composition between water and alcohol-consuming oxycodone rats, including: (1) 3 week total oxycodone intake clusters with Q_0_ and EPM locomotion; (2) Reinstatement Active Lever clusters with Log Alpha and P_max_ and (3) IL c-fos clusters with SSW. There are several similarities in cluster composition between male and female oxycodone + alcohol consuming rats including: 3 week ALC intake clusters with IL and NAc c-fos, while Log Alpha clusters with P_max_, Day 1 Extinction Active Lever, and CeA c-fos and 3 week oxycodone intake clusters with Q_0_, BLA and NAs c-fos. In contrast, oxycodone + water rats do not have many similarities in cluster formation between the sexes. For sucrose rats, approximately half of the variables remained in the same clusters. *3 wk Oxy intake* total oxycodone intake (mg/kg) in the first three weeks of IVSA (all FR-1, FR-3, FR-6, FR-10, FR-18); *3 wk ALC intake* total alcohol intake (g/kg) in the first three weeks of IVSA; *Time in OA* time spent in the open arms of the EPM; *EPM locomotion* total distance traveled in the EPM; *Day 1 Ext AL* Day 1 Extinction active lever presses. All other abbreviations defined in main text.

To gain insight into relationships between the many dependent variables recorded throughout the studies, we conducted MDS analysis. Clusters are depicted in different colors, with the color itself having no significance from panel to panel (Fig. 6). Shorter distances between variables indicate closer correlative relationships and vice versa. Under these analysis conditions, the MDS analysis ignores the direction relationships (i.e., positive vs. negative correlation), showing only where relationships exist. Pearson’s correlations found several correlated variables within each condition/sex (Supplementary Fig. S5). The optimal number of clusters for each condition was three, with the exception of female oxycodone + alcohol and sucrose + alcohol groups, which each had 4 clusters (Fig. 6). Cluster composition differed by condition. Relative to female oxycodone + water rats (Fig. 6a), male oxycodone + water rats (Fig. 6b) and female oxycodone + alcohol rats (Fig. 6d) display many differences in composition. Interestingly, female (Fig. 6d) and male oxycodone + alcohol rats (Fig. 6e) display several clusters with similar composition, including that alcohol intake clustered with NA core and IL c-fos expression, and in male rats only, this cluster also included SSW. In both male and female oxycodone + alcohol rats, oxycodone intake clusters with Q_0_, BLA and NAs c-fos expression. For male oxycodone + water and oxycodone + alcohol rats, P_max_ and log alpha clustered with reinstatement active lever presses, but this cluster was accompanied by distinct regions expressing c-fos for each group. Because sucrose rats have less variables included in the MDS (due to no demand curve analysis), we do not compare sucrose to oxycodone conditions. Alcohol-consuming sucrose rats displayed distinct cluster composition from water consuming rats, with almost no overlap in cluster composition (Fig. 6c,f).

## Discussion

Male and female rats permitted access to both oxycodone and alcohol decreased consumption of both drugs relative to rats having access to only one substance. Despite lower oxycodone intake, female PSU rats subsequently displayed reduced elasticity of demand for oxycodone that corresponded with increased cue-primed reinstatement of oxycodone-seeking. The opposite pattern was observed for male rats, with reduced reinstatement of oxycodone observed. Spontaneous withdrawal signs were correlated with oxycodone, and not alcohol, intake. Withdrawal from oxycodone was accompanied by reduced anxiety-like behavior in the EPM relative to oxycodone-naïve rats. Alcohol access increased the number of CeA neurons activated during cued sucrose-seeking and the number of BLA neurons activated by oxycodone-seeking in males only. Sex- and alcohol-dependent effects on activation of nucleus accumbens D1-expressing neurons were seen following both sucrose and oxycodone reinstatement tests. A data reductionist approach using multidimensional scaling followed by machine-learning based clustering revealed that both sex and alcohol access alter the relationship between key dependent variables. Thus, alcohol alters the motivation to seek oxycodone in a sex-dependent manner and alters the neural circuitry engaged by cue-primed reinstatement of sucrose and oxycodone-seeking.

Contrary to our hypotheses, the PSU condition reduced intake of oxycodone and alcohol relative to monosubstance conditions. While this could be due to cross-sensitization, only limited evidence supports this possibility; repeated morphine treatment results in cross-sensitization to the locomotor effects of alcohol, however the converse is not true^43^. The effects of non-contingent morphine on alcohol intake are biphasic, with low doses increasing voluntary alcohol intake and high doses decreasing intake; the decrease is blocked by naloxone, indicating that signaling through the mu opioid receptor underlies this effect^44^. It is possible that oxycodone-induced desensitization of the mu opioid receptor reduced motivation to consume alcohol, as systemic antagonism decreases alcohol consumption^45^. While such neurochemical adaptations may underlie the effects of oxycodone on alcohol intake, it’s less clear why alcohol would alter oxycodone intake on the following day. Alcohol access had no effect on sucrose intake, in agreement with prior work^46^, and ruling out generalized effects on motivation to seek rewards. It is possible that oxycodone altered the pharmacokinetics of alcohol to decrease intake. The half-life of intravenous oxycodone is approximately 62–83 min in the rat^47^, and thus was likely still present when rats were presented with alcohol. Thus, while there is evidence for alcohol–opioid interactions in the literature, particularly at the mu opioid receptor, the underlying pharmacokinetic and pharmacodynamic mechanisms behind the ability of alcohol to alter the motivation to seek oxycodone warrants further study.

While withdrawal from alcohol and oxycodone has the potential to produce SSW, no additive effects of the two drugs on spontaneous withdrawal were observed. Alcohol intake in sucrose rats did not increase SSW relative to the drug-naïve condition. Noncontingent alcohol and high levels of intake in mice (e.g., ≥ 10 g/kg/day) reliably produce SSW. Withdrawal signs are modest in models where blood alcohol levels (BALs) range from 150 to 200 mg%^18^. While we did not assess BAL here, the same alcohol access and range of consumption previously yielded BALs in the range of 50–80 mg%^23^. Thus, the present model likely does not engender high enough alcohol intake to produce SSW. We are the first to report that that SSW are positively correlated with intravenous oxycodone intake on the days prior to and after the assessment of SSW, in agreement with results following heroin IVSA^14^. Following oxycodone IVSA, measures of spontaneous withdrawal-related aggression and hyperalgesia have been reported 12 h after the last IVSA session^48^. Spontaneous SSW have also been observed following oral oxycodone intake in mice, when examined 24 h after the last oral intake session^49^.

Alcohol intake increased locomotion in the EPM but did not alter arm entries. Hyperlocomotion in a novel environment has been observed following alcohol consumption^50^. Acute oxycodone is anxiolytic^22^ and here we observe the same during short withdrawal from oxycodone (less than 24 h), potentially identifying anxiolysis as a factor underlying the motivation to consume both drugs. Sucrose is not anxiogenic in the present work, as this group’s EPM behavior is comparable to control (unstressed) rat behavior in other publications using the same procedures^51, 52^. Thus, despite being assessed at the same time point following the last self-administration session, oxycodone and oxycodone + alcohol have different effects on SSW and anxiety-like behavior, likely due to distinct neurochemical and neuroanatomical regulating these behaviors during withdrawal^53^. It is possible that additive effects of alcohol and oxycodone on these behaviors would be observed at other time points relative to the last drug exposure. Here, we chose to assess these behaviors at the same time that rats would have been placed into the operant chamber for the next self-administration session in order to determine whether withdrawal symptoms were related to the motivation to seek drug, finding that SSW were correlated with both past and future oxycodone intake.

While alcohol co-use decreased oxycodone intake during training in both sexes, in female rats, elasticity of demand for oxycodone was decreased by alcohol, indicating a reduced ability to alter behavior in the change of increased price (effort) necessary to obtain drug. Here, response requirements for oxycodone infusions increased, such that motivation to seek oxycodone was assessed when rats were largely undrugged (e.g., needed to emit lever presses to receive the first infusion) and thus acute pharmacological tolerance or sensitization does not explain these results. At this time, we cannot speculate on the underlying hormonal or neural substrates of the ability of alcohol to alter elasticity of demand for oxycodone only in females. Demand curve variables (P_max_, essential value, log α) that are measures of flexibility to increasing prices were correlated with active lever presses during the undrugged cue-primed reinstatement test, in agreement with similar work with cocaine and methamphetamine, and indicating that motivation to seek drug in both procedures are mechanistically linked^54, 55^. In females, opioid-alcohol co-consumption increased the motivation to seek oxycodone and the reinstatement of oxycodone-seeking. Alcohol did not alter demand elasticity in males, but cue-primed reinstatement of oxycodone-seeking did not occur when male rats had a history of consuming alcohol and oxycodone. However, it should be noted that there was an almost twofold increase in lever presses from extinction to test which did not attain statistical significance. Alcohol co-consumption did not alter the reinstatement of sucrose-seeking, indicating specificity for oxycodone-seeking. Alcohol consumption increases Q_0_ (preferred consumption when the price is zero) for intravenous cocaine, without affecting demand elasticity^56^.

Despite the relatively small sample size, several brain regions showed increased neuronal activation (i.e., number of c-fos^+^ cells) upon oxycodone reinstatement testing relative to untested rats: the NA core and shell, dSTR, BLA, CeA, and IL cortex. In the NA shell and dSTR, these increases were restricted to D1^+^ neurons, consistent with a role for these neuronal populations in driving drug-seeking^39^. The PL did not show a test-induced increase, but instead showed overall high expression in both tested and untested conditions, indicating the potential for lasting effects of oxycodone and alcohol intake on PL activity. Sex and alcohol-dependent changes in c-fos expression following oxycodone reinstatement were observed only in three regions: the NA core, NA shell, and BLA. In the NA shell, females in the oxycodone + alcohol group displayed the greatest amount of reinstatement-induced activity, specifically in D1^+^ cells, and in the NA core, females displayed increased c-fos expression overall, independent of alcohol history. Cocaine reinstatement-induced c-fos expression is greater in females than males in the NA core and shell, but only when the cocaine-cue pairings occurred during the estrus phase of the estrous cycle^57^. Here, reinforcer-cue pairings occurred throughout the estrous cycle and possibly contributed to greater cue-induced c-fos expression in females. Male oxycodone + alcohol rats displayed reduced test-induced c-fos expression in the NA core, and decreased reinstatement relative to other groups, indicating that this region may be impacted by PSU and be involved in mediating relapse. Active lever pressing during the cue test was negatively correlated with the number of c-fos^+^ cells in the BLA. A role for this brain region in the inhibition of reinstatement for any drug has not yet been reported in the literature, and in light of this region’s role in promoting cue-primed reinstatement, this correlation may have been due to increased activity of inhibitory interneurons that synapse onto the glutamatergic principal neurons that drive reinstatement^36^. Taken together, unlike the profound effects of alcohol on cocaine reinstatement-induced c-fos expression observed in these regions in a similar sequential model of cocaine-alcohol PSU, only modest effects of PSU were observed here. Thus, it may be that relapse to oxycodone seeking engages similar circuitries in the presence and absence of alcohol consumption.

A history of alcohol consumption increased the number of CeA neurons activated during cued oxycodone – and sucrose-seeking, and the number of ventral and dorsal striatum D1-expressing neurons engaged by sucrose reinstatement, without corresponding increases in reinstatement. Reinstatement-induced c-fos expression may be enhanced by increased baseline synaptic activity following a history of alcohol intake. Increased glutamate transmission and/or decreased GABA interneuron activity in the BLA and dorsal and ventral striatum occur following alcohol exposure^58–61^. The small number of sucrose rats in the untested condition did not permit assessment of the long-term effects of alcohol alone on c-fos expression. However, following 2 months of IAA, male and female Sprague–Dawley rats display no changes in NA core, NA shell, CeA and BLA c-fos expression at 28 days withdrawal, relative to alcohol-naïve rats^62^. Thus, it is likely that the interaction between alcohol and the cue test increased c-fos expression in the present work.

We observed no sex differences in oxycodone intravenous self-administration, consistent with reports in Sprague Dawley and other rat strains^47, 63^, with the exception of female Wistars which self-administer a greater number of infusions than males^48^. Despite no differences in oxycodone intake, increased cued seeking after two^63^, but not four weeks of home cage abstinence^47^ has been reported in Sprague Dawley females relative to males. Here, sex-differences in cued oxycodone seeking were only observed in the alcohol condition. In summary, sex differences in oxycodone intake and cued seeking are dependent on the strain of rat and addiction model used.

MDS analysis revealed that both sex and access to alcohol altered cluster composition, i.e. the relationship between dependent variables. There are a greater number of differences in cluster composition between female oxycodone + water and female oxycodone + alcohol rats than similarities. For male and female oxycodone + alcohol consuming rats, there were many similarities in cluster composition, while the same is not true for oxycodone + water rats. While inferential statistics reveal no differences between oxycodone + water and oxycodone + alcohol male rats in terms of demand parameters and c-fos expression in many brain regions, MDS-based clustering finds distinct clusters in these conditions, with the BLA, NA core and shell c-fos clustering with active lever presses during reinstatement in the oxycodone + water males, and the dSTR, PL and CeA c-fos expression clustering with this behavior in the oxycodone + alcohol males. It may be that alcohol intake itself caused the differences in cluster composition, however, alcohol also influenced other dependent variables (e.g., oxycodone intake), which may in turn alter the relationships between variables. These findings highlight the importance of utilizing multiple analysis approaches to decipher complex relationships, including those involving multiple drugs and sexes.

## Conclusions

Alcohol consumption reduces oxycodone intake during self-administration in both sexes, while altering the motivation to seek oxycodone in a sex-dependent manner. Inferential statistics found that alcohol-oxycodone PSU has modest effects on the neural circuitry engaged by cue-primed reinstatement of oxycodone-seeking, with the NA shell uniquely showing expression that mapped onto behavior during the test. While the sample size for each sex was small for the reinstatement and c-fos analyses, the reinstatement data is correlated with demand elasticity, indicating that the smaller sample size was representative of a larger group. The MDS analysis found that alcohol intake altered the relationship between oxycodone-seeking variables (from intake to economic demand and reinstatement), c-fos expression, anxiety-like behavior and withdrawal signs. Sex also had an impact on such relationships. These results build upon our prior work finding that sequential cocaine-alcohol self-administration alters the role of accumbens glutamate release in cocaine reinstatement as well as reinstatement-induced c-fos protein expression throughout the reward circuitry^23, 32^. Future work examining pharmacological interactions between alcohol and oxycodone are necessary to interpret the ability of these drugs to influence the intake of one another. These data, together with the clinical significance of PSU, strongly indicate that more research is needed to characterize the behavioral and neurobiological effects of PSU in order to develop targeted therapies for reducing drug use.

### Supplementary Information

Supplementary Information.

## Data Availability

Data will be made available upon request to the corresponding author following publication.

## References

[1] Falk D, Yi H, Hiller-Sturmhöfel S (2008). An epidemiologic analysis of co-occurring alcohol and drug use and disorders: Findings from the National Epidemiologic Survey of Alcohol and Related Conditions (NESARC). Alcohol Res. Health..

[2] Rhee TG, Rosenheck RA (2019). Association of current and past opioid use disorders with health-related quality of life and employment among US adults. Drug Alcohol Depend..

[3] Schepis TS, Hakes JK (2017). Age of initiation, psychopathology, and other substance use are associated with time to use disorder diagnosis in persons using opioids nonmedically. Subst. Abus..

[4] Roux P, Lions C, Michel L, Cohen J, Mora M, Marcellin F (2014). Predictors of non-adherence to methadone maintenance treatment in opioid-dependent individuals: Implications for clinicians. Curr. Pharm. Des..

[5] Hickman M, Lingford-Hughes A, Bailey C, Macleod J, Nutt D, Henderson G (2008). Does alcohol increase the risk of overdose death: The need for a translational approach. Addiction.

[6] Jones CM, Paulozzi LJ, Mack KA, Centers for Disease Control and Prevention (CDC) (2014). Alcohol involvement in opioid pain reliever and benzodiazepine drug abuse-related emergency department visits and drug-related deaths - United States, 2010. MMWR Morb. Mortal Wkly. Rep..

[7] Levine B, Green D, Smialek JE (1995). The role of ethanol in heroin deaths. J. Forensic Sci..

[8] Zacny JP, Gutierrez S (2011). Subjective, psychomotor, and physiological effects of oxycodone alone and in combination with ethanol in healthy volunteers. Psychopharmacology.

[9] Wilson, N., Kariisa, M., Puja, S., Smith, H. & Davis, N. *Drug and Opioid-Involved Overdose Deaths—United States, 2017–2018* (CDC, 2020). https://stacks.cdc.gov/view/cdc/99464. Accessed March 9, 2021. 10.15585/mmwr.mm6911a4 PMC7739981 32191688

[10] Kalkman GA, Kramers C, van Dongen RT, van den Brink W, Schellekens A (2019). Trends in use and misuse of opioids in the Netherlands: A retrospective, multi-source database study. Lancet Public Health.

[11] Peacock A, Bruno R, Larance B, Lintzeris N, Nielsen S, Ali R (2016). Same-day use of opioids and other central nervous system depressants amongst people who tamper with pharmaceutical opioids: A retrospective 7-day diary study. Drug Alcohol Depend..

[12] Larance B, Campbell G, Peacock A, Nielsen S, Bruno R, Hall W (2016). Pain, alcohol use disorders and risky patterns of drinking among people with chronic non-cancer pain receiving long-term opioid therapy. Drug Alcohol. Depend..

[13] Torres-Lockhart KE, Lu TY, Weimer MB, Stein MR, Cunningham CO (2022). Clinical management of opioid withdrawal. Addiction.

[14] Gipson CD, Dunn KE, Bull A, Ulangkaya H, Hossain A (2021). Establishing preclinical withdrawal syndrome symptomatology following heroin self-administration in male and female rats. Exp. Clin. Psychopharmacol..

[15] Carper M, Contreras KM, Walentiny DM, Beardsley PM, Damaj MI (2021). Validation and characterization of oxycodone physical dependence in C57BL/6J mice. Eur. J. Pharmacol..

[16] Moussawi K, Ortiz MM, Gantz SC, Tunstall BJ, Marchette RCN, Bonci A (2020). Fentanyl vapor self-administration model in mice to study opioid addiction. Sci. Adv..

[17] Bobzean SAM, Kokane SS, Butler BD, Perrotti LI (2019). Sex differences in the expression of morphine withdrawal symptoms and associated activity in the tail of the ventral tegmental area. Neurosci. Lett..

[18] Vendruscolo LF, Roberts AJ (2014). Operant alcohol self-administration in dependent rats: Focus on the vapor model. Alcohol.

[19] Bloodgood DW, Hardaway JA, Stanhope CM, Pati D, Pina MM, Neira S (2021). Kappa opioid receptor and dynorphin signaling in the central amygdala regulates alcohol intake. Mol. Psychiatry.

[20] Neira S, Hassanein LA, Stanhope CM, Buccini MC, D’Ambrosio SL, Flanigan ME (2022). Chronic alcohol consumption alters home-cage behaviors and responses to ethologically relevant predator tasks in mice. Alcohol. Clin. Exp. Res..

[21] Vranjkovic O, Winkler G, Winder DG (2018). Ketamine administration during a critical period after forced ethanol abstinence inhibits the development of time-dependent affective disturbances. Neuropsychopharmacology.

[22] Bruijnzeel AW, Behnood-Rod A, Malphurs W, Chellian R, Caudle RM, Febo M (2022). Oxycodone decreases anxiety-like behavior in the elevated plus-maze test in male and female rats. Behav. Pharmacol..

[23] Stennett BA, Padovan-Hernandez Y, Knackstedt LA (2020). Sequential cocaine-alcohol self-administration produces adaptations in rat nucleus accumbens core glutamate homeostasis that are distinct from those produced by cocaine self-administration alone. Neuropsychopharmacology.

[24] Vigneault É (2015). Distribution of vesicular glutamate transporters in the human brain. Front. Neuroanat..

[25] Rubio FJ, Quintana-Feliciano R, Warren BL, Li X, Witonsky KFR, Valle FSD (2019). Prelimbic cortex is a common brain area activated during cue-induced reinstatement of cocaine and heroin seeking in a polydrug self-administration rat model. Eur. J. Neurosci..

[26] Mashhoon Y, Wells AM, Kantak KM (2010). Interaction of the rostral basolateral amygdala and prelimbic prefrontal cortex in regulating reinstatement of cocaine-seeking behavior. Pharmacol. Biochem. Behav..

[27] LaLumiere RT, Kalivas PW (2008). Glutamate release in the nucleus accumbens core is necessary for heroin seeking. J Neurosci..

[28] Li X, Zeric T, Kambhampati S, Bossert JM, Shaham Y (2015). The central amygdala nucleus is critical for incubation of methamphetamine craving. Neuropsychopharmacology.

[29] O’Neal TJ, Nooney MN, Thien K, Ferguson SM (2020). Chemogenetic modulation of accumbens direct or indirect pathways bidirectionally alters reinstatement of heroin-seeking in high- but not low-risk rats. Neuropsychopharmacology.

[30] Roura-Martínez D, Ucha M, Orihuel J, Ballesteros-Yáñez I, Castillo CA, Marcos A (2020). Central nucleus of the amygdala as a common substrate of the incubation of drug and natural reinforcer seeking. Addict. Biol..

[31] Heinsbroek JA, Giannotti G, Mandel MR, Josey M, Aston-Jones G, James MH (2021). A common limiter circuit for opioid choice and relapse identified in a rodent addiction model. Nat. Commun..

[32] Stennett BA, Knackstedt LA (2020). A rat model of cocaine-alcohol polysubstance use reveals altered cocaine seeking and glutamate levels in the nucleus accumbens. Front. Neurosci..

[33] Altshuler RD, Yang ES, Garcia KT, Davis IR, Olaniran A, Haile M (2021). Role of orbitofrontal cortex in incubation of oxycodone craving in male rats. Addict. Biol..

[34] McReynolds JR, Christianson JP, Blacktop JM, Mantsch JR (2018). What does the Fos say? Using Fos-based approaches to understand the contribution of stress to substance use disorders. Neurobiol. Stress.

[35] Burma NE, Bonin RP, Leduc-Pessah H, Baimel C, Cairncross ZF, Mousseau M (2017). Blocking microglial pannexin-1 channels alleviates morphine withdrawal in rodents. Nat. Med..

[36] Stefanik MT, Kalivas PW (2013). Optogenetic dissection of basolateral amygdala projections during cue-induced reinstatement of cocaine seeking. Front. Behav. Neurosci..

[37] Stefanik MT, Moussawi K, Kupchik YM, Smith KC, Miller RL, Huff ML (2013). Optogenetic inhibition of cocaine seeking in rats. Addict. Biol..

[38] Augur IF, Wyckoff AR, Aston-Jones G, Kalivas PW, Peters J (2016). Chemogenetic activation of an extinction neural circuit reduces cue-induced reinstatement of cocaine seeking. J. Neurosci..

[39] Bock R, Shin JH, Kaplan AR, Dobi A, Markey E, Kramer PF (2013). Strengthening the accumbal indirect pathway promotes resilience to compulsive cocaine use. Nat. Neurosci..

[40] Hout MC, Papesh MH, Goldinger SD (2013). Multidimensional scaling. Wiley Interdiscip. Rev. Cogn. Sci..

[41] Hursh SR, Silberberg A (2008). Economic demand and essential value. Psychol. Rev..

[42] Hursh SR, Roma PG (2016). Behavioral economics and the analysis of consumption and choice. Manage Decis. Econ..

[43] Lessov CN, Phillips TJ (2003). Cross-sensitization between the locomotor stimulant effects of ethanol and those of morphine and cocaine in mice. Alcohol. Clin. Exp. Res..

[44] Vacca G, Serra S, Brunetti G, Carai MAM, Gessa GL, Colombo G (2002). Boosting effect of morphine on alcohol drinking is suppressed not only by naloxone but also by the cannabinoid CB1 receptor antagonist, SR 141716. Eur. J. Pharmacol..

[45] Minnaard AM, Ramakers GMJ, Vanderschuren LJMJ, Lesscher HMB (2021). Baclofen and naltrexone, but not N-acetylcysteine, affect voluntary alcohol drinking in rats regardless of individual levels of alcohol intake. Behav. Pharmacol..

[46] Pastor R, Kamens HM, McKinnon CS, Ford MM, Phillips TJ (2010). Repeated ethanol administration modifies the temporal structure of sucrose intake patterns in mice: Effects associated with behavioral sensitization. Addict. Biol..

[47] Doyle MR, Martinez AR, Qiao R, Dirik S, Di Ottavio F, Pascasio G (2023). Strain and sex-related behavioral variability of oxycodone dependence in rats. Neuropharmacology.

[48] Kimbrough A, Kononoff J, Simpson S, Kallupi M, Sedighim S, Palomino K (2020). Oxycodone self-administration and withdrawal behaviors in male and female Wistar rats. Psychopharmacology.

[49] McKendrick G, McDevitt DS, Shafeek P, Cottrill A, Graziane NM (2022). Anterior cingulate cortex and its projections to the ventral tegmental area regulate opioid withdrawal, the formation of opioid context associations and context-induced drug seeking. Front. Neurosci..

[50] Rasmussen DD, Mitton DR, Green J, Puchalski S (2001). Chronic daily ethanol and withdrawal: 2. Behavioral changes during prolonged abstinence. Alcohol Clin. Exp. Res..

[51] Schwendt M, Shallcross J, Hadad NA, Namba MD, Hiller H, Wu L (2018). A novel rat model of comorbid PTSD and addiction reveals intersections between stress susceptibility and enhanced cocaine seeking with a role for mGlu5 receptors. Transl. Psychiatry..

[52] Blount HL, Dee J, Wu L, Schwendt M, Knackstedt LA (2023). Stress resilience-associated behaviors following predator scent stress are accompanied by upregulated nucleus accumbens mGlu5 transcription in female Sprague Dawley rats. Behav. Brain Res..

[53] Monroe SC, Radke AK (2023). Opioid withdrawal: Role in addiction and neural mechanisms. Psychopharmacology.

[54] Bentzley BS, Jhou TC, Aston-Jones G (2014). Economic demand predicts addiction-like behavior and therapeutic efficacy of oxytocin in the rat. Proc. Natl. Acad. Sci. USA.

[55] Galuska CM, Banna KM, Willse LV, Yahyavi-Firouz-Abadi N, See RE (2011). A comparison of economic demand and conditioned-cued reinstatement of methamphetamine-seeking or food-seeking in rats. Behav. Pharmacol..

[56] James MH, Fragale JE, O’Connor SL, Zimmer BA, Aston-Jones G (2021). The orexin (hypocretin) neuropeptide system is a target for novel therapeutics to treat cocaine use disorder with alcohol coabuse. Neuropharmacology.

[57] Johnson AR, Thibeault KC, Lopez AJ, Peck EG, Sands LP, Sanders CM (2019). Cues play a critical role in estrous cycle-dependent enhancement of cocaine reinforcement. Neuropsychopharmacology.

[58] McGinnis MM, Parrish BC, Chappell AM, Alexander NJ, McCool BA (2020). Chronic ethanol differentially modulates glutamate release from dorsal and ventral prefrontal cortical inputs onto rat basolateral amygdala principal neurons. ENeuro.

[59] Pati D, Kelly K, Stennett B, Frazier CJ, Knackstedt LA (2016). Alcohol consumption increases basal extracellular glutamate in the nucleus accumbens core of Sprague-Dawley rats without increasing spontaneous glutamate release. Eur. J. Neurosci..

[60] Klenowski PM, Fogarty MJ, Drieberg-Thompson JR, Bellingham MC, Bartlett SE (2021). Reduced inhibitory inputs on basolateral amygdala principal neurons following long-term alcohol consumption. Neuroscience.

[61] Wilcox MV, Cuzon Carlson VC, Sherazee N, Sprow GM, Bock R, Thiele TE (2014). Repeated binge-like ethanol drinking alters ethanol drinking patterns and depresses striatal GABAergic transmission. Neuropsychopharmacology.

[62] Li J, Chen P, Han X, Zuo W, Mei Q, Bian EY (2019). Differences between male and female rats in alcohol drinking, negative affects and neuronal activity after acute and prolonged abstinence. Int. J. Physiol. Pathophysiol. Pharmacol..

[63] Guha SK, Alonso-Caraballo Y, Driscoll GS, Babb JA, Neal M, Constantino NJ (2022). Ranking the contribution of behavioral measures comprising oxycodone self-administration to reinstatement of drug-seeking in male and female rats. Front. Behav. Neurosci..

