# Supplementary material for: Voluntary alcohol intake alters the motivation to seek intravenous oxycodone and neuronal activation during the reinstatement of oxycodone and sucrose seeking
Source: Sci Rep. 2023 Nov 6;13:19174. doi: 10.1038/s41598-023-46111-1 (PMC10628226; doi:10.1038/s41598-023-46111-1)
Supplement: Supplementary file 1 — Supplementary Information. [file 41598_2023_46111_MOESM1_ESM.pdf]

## Supplementary Data

**Supplementary Table S1**. Mean and SEM for demand curve variables.

| Dependent Variable | Oxy+ALC female | | Oxy+$H_2O$ female | | Oxy+ALC male | | Oxy+$H_2O$ male | |
|---|---|---|---|---|---|---|---|---|
| | mean | SEM | mean | SEM | mean | SEM | mean | SEM |
| **$Q_0$** | 38.60 | 9.99 | 49.74 | 11.57 | 35.49 | 6.14 | 48.22 | 13.40 |
| **Pmax** | 78.40 | 28.67 | 44.35 | 7.06 | 34.35 | 9.08 | 50.38 | 11.49 |
| **α (log)** | -4.054 | 0.523 | -3.819 | 0.465 | -4.115 | 0.330 | -4.076 | 0.502 |
| **Essential Value** | 30.32 | 15.75 | 21.55 | 7.07 | 12.68 | 3.76 | 24.73 | 7.84 |

**Supplementary Table S2.** Results of 3-way Sex x Liquid x Test ANOVAs for c-fos expression in Oxy rats.

| **Dependent Variable** | **Main effect of Test** | | **Sex x Liquid x Test** | | **Liquid x Test** | | **Sex x Test** | |
|---|---|---|---|---|---|---|---|---|
| | F stat | p-value | F stat | p-value | F stat | p-value | F stat | p-value |
| IL # c-fos$^+$/vGlut1$^+$ cells | 11.39 | **<0.05*** | 0.52 | n.s | 0.04 | n.s | 1.48 | n.s |
| PL # c-fos+/vGlut1+ cells | 3.04 | n.s | 0.78 | n.s | 0.19 | n.s | 0.55 | n.s |
| NAc # c-fos$^+$/D1$^+$ cells | 28.49 | **<0.001*** | 0.15 | n.s | 0.04 | n.s | 1.04 | n.s |
| NAs # c-fos$^+$/D1$^+$ cells | 17.56 | **<0.001*** | 1.75 | n.s | 0.12 | n.s | 0.18 | n.s |
| dSTR # c-fos$^+$/D1$^+$ cells | 11.27 | **<0.01*** | 3.07 | n.s | 0.55 | n.s | 0.20 | n.s |
| | | | | | | | | |
| IL # c-fos$^+$ cells | 6.03 | **<0.05*** | 1.48 | n.s | 0.02 | n.s | 0.87 | n.s |
| PL # c-fos+ cells | 2.03 | n.s | 0.49 | n.s | 0.01 | n.s | 0.45 | n.s |
| BLA # c-fos$^+$ cells | 99.55 | **<0.05*** | 2.46 | n.s | 4.80 | **<0.05*** | 0.11 | n.s |
| CeA # c-fos$^+$ cells | 19.802 | **<0.001*** | 0.77 | n.s. | 0.028 | n.s. | 6.661 | **<0.05*** |
| NAc # c-fos$^+$ cells | 35.58 | **<0.05*** | 0.02 | n.s | 0.37 | n.s | 0.83 | n.s |
| NAs # c-fos$^+$ cells | 3.31 | n.s | 2.16 | n.s | 0.03 | n.s | 0.01 | n.s |
| dSTR # c-fos$^+$ cells | 2.63 | n.s | 1.71 | n.s | 0.002 | n.s | 0.01 | n.s |

## Supplementary Figures

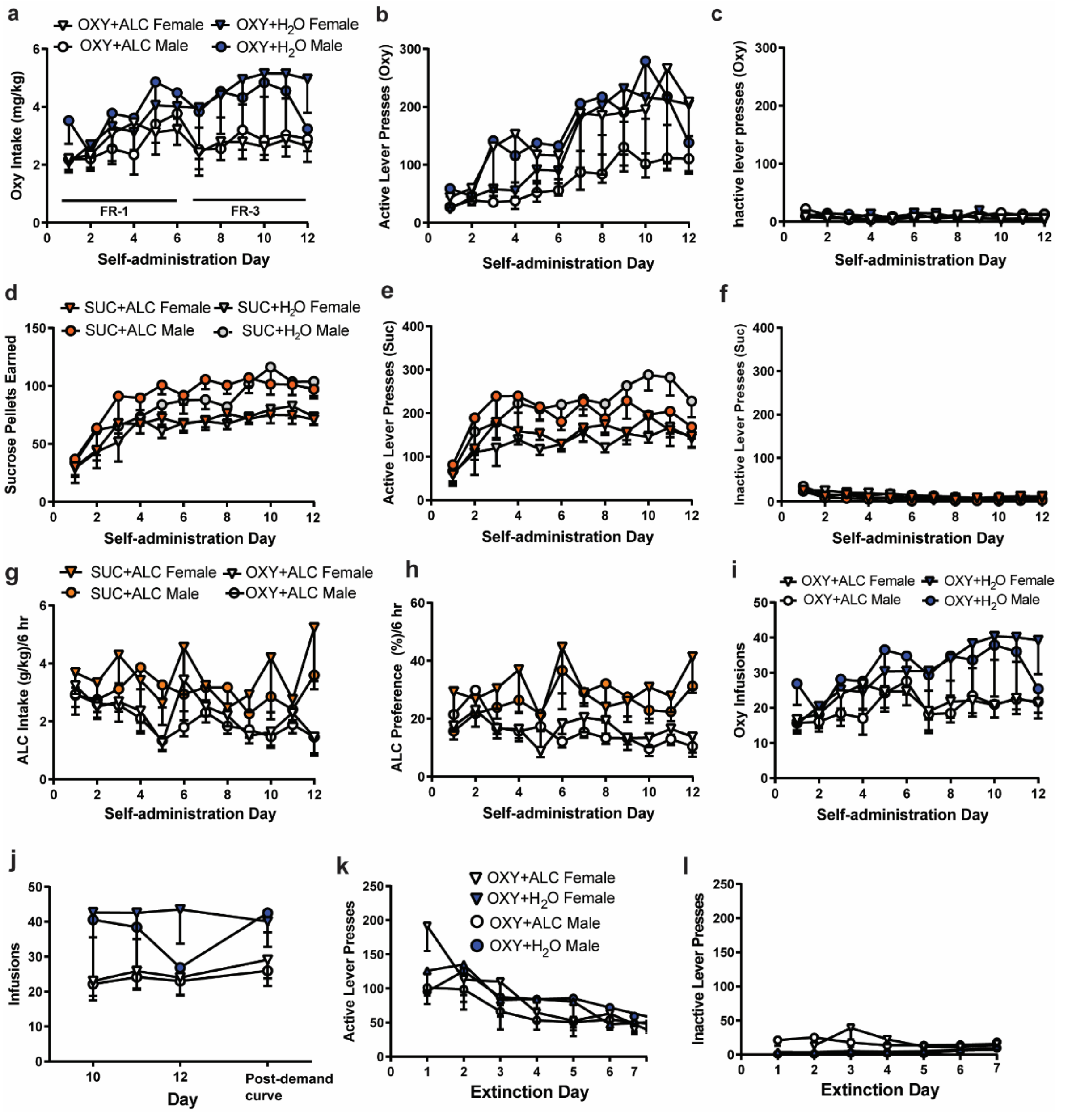

**Supplementary Fig. S1.** There were no effects of sex on oxycodone intake (**a**), active lever presses (**b**), or inactive lever presses (**c**). There were no effects of sex on sucrose intake (**d**), active lever presses (**e**), or inactive lever presses (**f**). There were no sex differences in alcohol intake (**g**) or preference (**h**). **i.** Alcohol decreased the number of oxycodone infusions with no effect of sex [$F_{(11, 341)}$=2.406, p<0.01]. There were no effects of sex on oxycodone infusions during self-administration when the last three days of self-administration prior to the demand curve were compared to infusions earned on an FR-3 schedule after the demand curve **(j).** There were no effects of sex on active (**k**) or inactive lever presses (**l**) during extinction training.

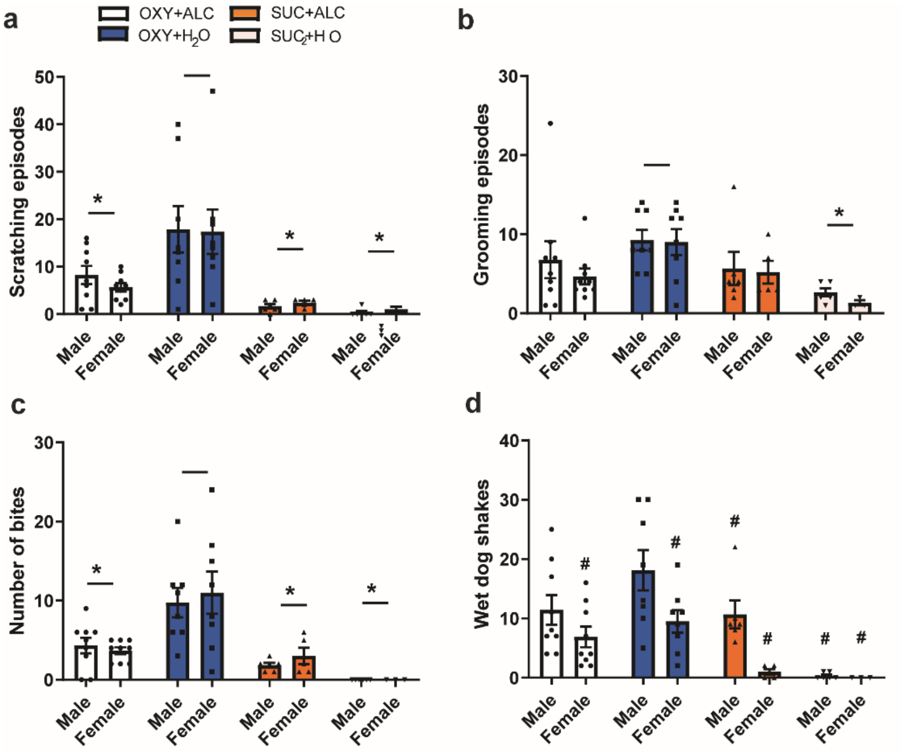


Supplementary Fig. S2. Individual somatic signs of withdrawal. Examining withdrawal signs individually revealed similar patterns as the global withdrawal score. There were significant Reinforcer x Liquid interactions for (**a**) scratching episodes [$F_{(1,47)}$ = 5.424, p= 0.024], (**b**) grooming episodes [$F_{(1,47)}$ = 6.114, p= 0.017], and (**c**) biting [$F_{(1,47)}$ = 11.782, p= 0.001], For both scratching and biting, the OXY+$H_2O$ rats displayed greater numbers than all other groups. For grooming, the OXY+$H_2O$ rats displayed greater number of episodes only relative to the SUC+$H_2O$. **d.** There was a significant Sex x Reinforcer interaction for the number of wet dog shakes [$F_{(1,47)}$ = 4.356, p= 0.042], with male OXY rats exhibiting a greater number than all other conditions.

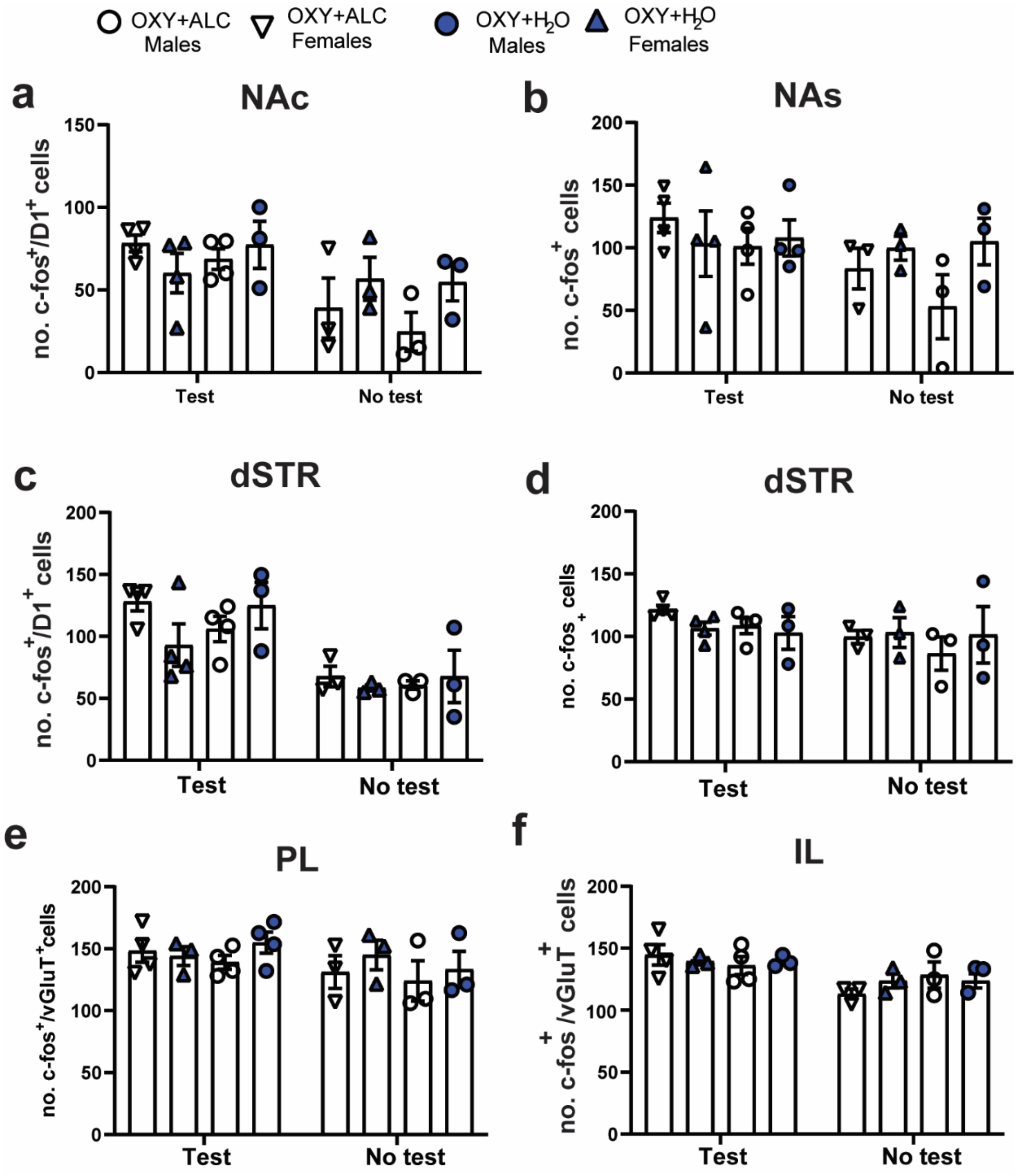


**Supplementary Fig. S3**. **a.** There was a main effect of Test on c-fos expression in D1$^{+}$ cells of the NAc, but no effects of Liquid and a trend for an effect of Sex (p=0.07) on expression. **b.** There was no effect of Test on c-fos$^{+}$ cells in the NAs. **c**. There was a main effect of Test for c-fos expression in D1$^{+}$ cells of the dSTR, expression did not differ by Sex or Liquid. **d**. There was no effect of Test on c-fos$^{+}$ cells in the dSTR. **e**. There was no effect of Test on c-fos expression in vGlut1$^{+}$ cells of the PL. **f.** There was a main effect of Test for c-fos expression in vGlut1$^{+}$ cells of the IL, but no effects of Liquid or Sex on expression.

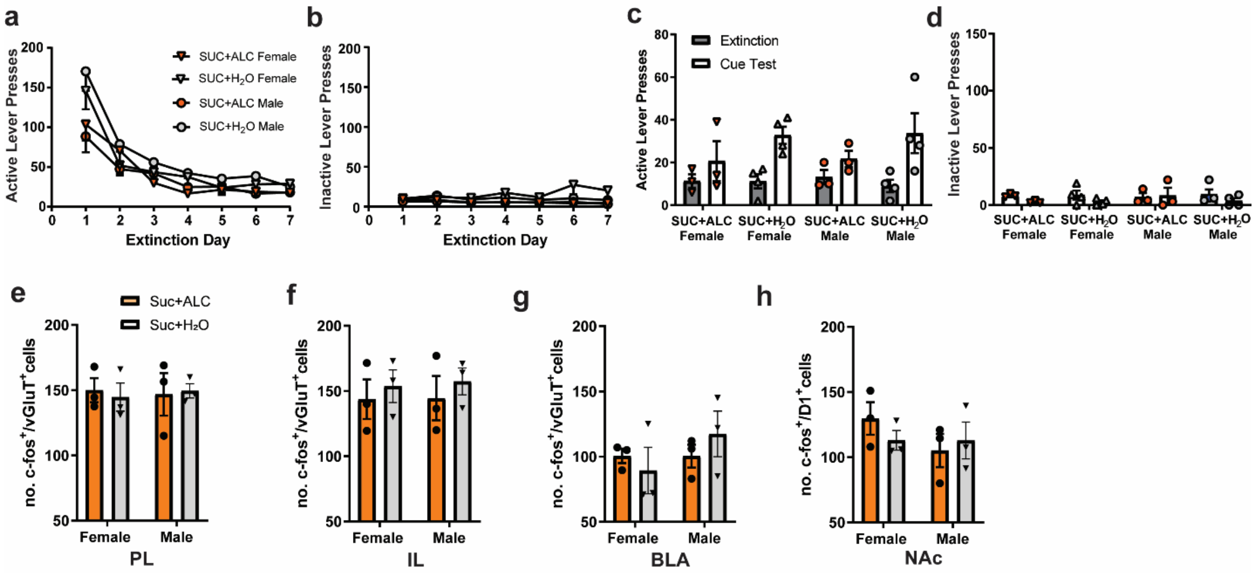


**Supplementary Fig. S4.** There were no effects of sex on active lever presses (**a**), or inactive lever presses (**b**) during extinction training following sucrose self-administration. There were no effects of sex on active lever presses (**c**), or inactive lever presses (**d**) when comparing the last day of extinction training to the cue test. Alcohol did not alter c-fos expression following the sucrose cue-test in male or female rats in glutamate neurons of the PL (**e**), IL **(f)**, BLA (**g**), or in D1-expressing neurons in the NAc (**h**).

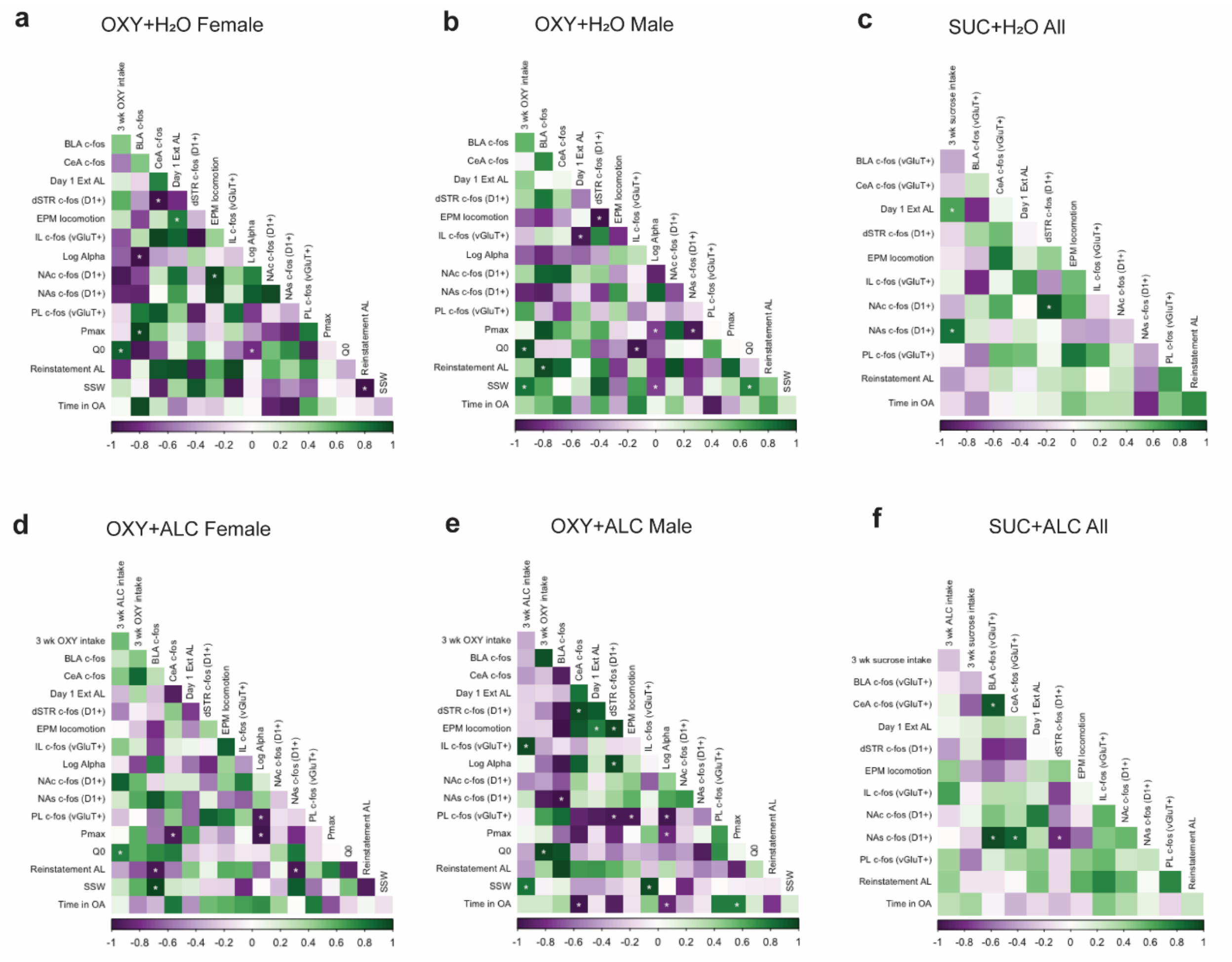


**Supplementary Fig. 5.** Pearson's correlations for MDS variables. N's = 4-9/sex/condition. * = $p<0.05$
